# VHL mutation-mediated SALL4 overexpression promotes tumorigenesis and vascularization of clear cell renal cell carcinoma via Akt/GSK-3β signaling

**DOI:** 10.1186/s13046-020-01609-8

**Published:** 2020-06-08

**Authors:** Jinbo Sun, Qisheng Tang, Yongheng Gao, Wei Zhang, Zhining Zhao, Fan Yang, Xiangnan Hu, Dan Zhang, Yong Wang, Huizhong Zhang, Bin Song, Bo Zhang, He Wang

**Affiliations:** 1 Department of Urology, Tangdu Hospital, Fourth Military Medical University, Xi’an, 710038 Shaanxi China; 2 Department of Respiratory and Critical Care Medicine, Tangdu Hospital, Fourth Military Medical University, Xi’an, 710038 Shaanxi China; 3 Clinical Laboratory, The 986th Military Hospital, Fourth Military Medical University, Xi’an, 710054 Shaanxi China; 4 Department of Pathology, Xi’an No. 3 Hospital, The Affiliated Hospital of Northwest University, Xi’an, 710018 Shaanxi China; 5 Medical Laboratory and Research Center, Tangdu Hospital, Fourth Military Medical University, Xi’an, 710038 Shaanxi China

**Keywords:** Clear cell renal cell carcinoma, SALL4, Tumorigenesis, Angiogenesis, VHL mutation

## Abstract

**Background:**

Although ongoing development of therapeutic strategies contributes to the improvements in clinical management, clear cell renal cell carcinoma (ccRCC) deaths originate mainly from radiochemoresistant and metastatic disease. Transcription factor SALL4 has been implicated in tumorigenesis and metastasis of multiple cancers. However, it is not known whether SALL4 is involved in the pathogenesis of ccRCC.

**Methods:**

Analyses of clinical specimen and publicly available datasets were performed to determine the expression level and clinical significance of SALL4 in ccRCC. The influence of SALL4 expression on ccRCC tumor growth, metastasis and vascularity was evaluated through a series of in vitro and in vivo experiments. Western blotting, immunofluorescence staining and integrative database analysis were carried out to investigate the underlying mechanism for SALL4-mediated oncogenic activities in ccRCC.

**Results:**

SALL4 expression was increased in ccRCC and positively correlated with tumor progression and poor prognosis. SALL4 could promote ccRCC cell proliferation, colony formation, cell cycle progression, migration, invasion and tumorigenicity and inhibit cell senescence. Further investigation revealed a widespread association of SALL4 with individual gene transcription and the involvement of SALL4 in endothelium development and vasculogenesis. In the context of ccRCC, SALL4 promoted tumor vascularization by recruiting endothelial cells. In addition, we found that SALL4 could exert its tumor-promoting effect via modulating Akt/GSK-3β axis and VEGFA expression. VHL mutation and DNA hypomethylation may be involved in the upregulation of SALL4 in ccRCC.

**Conclusions:**

Overall, our results provide evidence that upregulated SALL4 can function as a crucial regulator of tumor pathogenesis and progression in ccRCC, thus offering potential therapeutic strategies for future treatment.

## Background

Renal cell carcinoma, stemming from the renal tubular epithelium, is one of the top 10 leading malignancies. As a most frequent subtype of renal cell carcinoma, clear cell renal cell carcinoma (ccRCC) comprises approximately 70% of kidney cancers. Development of metastatic spread and radiochemoresistance contributes to a poor prognosis, as evidenced by a dismal 8–12% five-year overall survival of metastatic ccRCC patients [1]. It is universally acknowledged that ccRCC is a highly vascularized malignancy and therapies targeting angiogenesis are initially efficacious in tumor regression. Unfortunately, it is inevitable that acquisition of drug resistance occurs within a year [2] and renders this treatment invalid in most patients. Significant efforts have been devoted to elucidate the molecular dependencies and vulnerabilities of ccRCC and patients who fail to respond to conventional treatments are in urgent need for new therapeutic strategies.

Mutation of the von Hippel-Lindau (VHL) tumor suppressor is observed in roughly 80% of ccRCC tumors and identified as one of the genetic determinants driving ccRCC initiation and progression [3]. It is well known that VHL, a component of the E3 ubiquitin ligase complex, functions as a negative regulator of hypoxia-inducible factor (HIF) signaling by targeting HIF-1/2α. Inactivated mutation of VHL in ccRCC frees HIF-1/2α from VHL-mediated ubiquitination and degradation [4]. As a result, accumulated HIF-1/2α drives transcriptional activation of its downstream target genes related to metabolism, cell cycle and angiogenesis [5, 6], which contributes to ccRCC development. Extensive crosstalk has been reported to exist between PI3K/Akt/mTOR and VHL/HIF pathways [7]. VHL loss-mediated HIF overexpression results in transactivation of multiple growth factors, such as vascular endothelial growth factor (VEGF) that can promote activation of PI3K/Akt/mTOR signaling [8], and PI3K/Akt pathway in turn can drives the transcription of HIF and its target genes via mTOR activation. These findings support the observation that PI3K/Akt/mTOR pathway is aberrantly activated and established as a promising drug target for ccRCC, which has yielded a efficacious therapy targeting mTOR for treatment of metastatic ccRCC [9].

Transcription factor SALL4 (sal-like 4), abundantly expressed in fetal tissues, is identified as a stemness factor that is involved in embryonic stem cell pluripotency and embryonic development [10]. Restored SALL4 expression has been reported to be detectable in various tumors and exhibit oncogenic roles in cancer genesis and progression. Recent evidence has indicated that SALL4, exhibiting progression-relevant expression, drives tumorigenesis, metastasis and radiochemotherapy resistance in gastric cancer, nasopharyngeal carcinoma and hepatocellular carcinoma [11–13]. Furthermore, elevated SALL4 expression highly correlated with worse overall survival [14]. Therapeutic peptides targeting SALL4 exhibit potent antitumor activity in hepatocellular carcinoma [13, 15], thus establishing SALL4 as a promising drug target. However, the roles of SALL4 in ccRCC tumorigenesis and progression remain poorly understood.

In this study, we explored the biologic roles and mechanisms governed by SALL4 in the pathogenesis of ccRCC. We found that upregulated SALL4 in ccRCC positively correlated with tumor progression. Our data indicated that SALL4 downregulation attenuated ccRCC tumor growth, metastasis and angiogenesis. We further demonstrated that knockdown of SALL4 conduced to a decrease in phosphoprotein markers of PI3K/Akt pathway activation including p-Akt and p-GSK-3β, as well as decreased VEGFA levels. A mechanistic link between VHL mutation and SALL4 upregulation was observed in ccRCC. Therefore, this work provides strong evidence that SALL4 is functionally important in ccRCC progression and may be a promising drug target.

## Methods

### Cell culture

The human ccRCC cell lines (ACHN, 786-O) and human umbilical vein endothelial cell (HUVEC) were cultured in RPMI 1640 (786-O) or DMEM/F-12 (ACHN, HUVEC) medium (Gibco) containing 10% fetal bovine serum (FBS, Bioind) and 1% penicillin/streptomycin (Gibco). All the cell lines were maintained in an incubator (37 °C) containing 5% (v/v) humidified CO_2_.

### Lentivirus transduction

Stable SALL4 knockdown cells were established by lentiviral shRNA infection. The lentiviral particles carrying shRNA against SALL4 (shSALL4, sh#1 and sh#2) or negative control (shNC) were generated and provided by Obio Technology (Shanghai, China). The cells (ACHN, 786-O) were transduced with lentivirus following the manufacturer’s instructions. To establish stable cell lines, the infected cells were treated with puromycin (2 μg/mL) for 7 days. The shRNA sequences for SALL4: GCCTTGAAACAAGCCAAGCTA (sh#1) and GAGGATGAAGCCACAGTAA (sh#2); the shRNA sequence for negative control: TTCTCCGAACGTGTCACGT (shNC).

### Cell counting Kit-8 (CCK-8) assay

Cells (1 × 10^3^ cells/well) were seeded in 96-well plates and maintained for indicated time. Cell growth was monitored by incubation with CCK-8 solution (Sangon Biotech) following the manufacturer’s protocols. Then a microplate reader (Bio-Rad) was used to detect absorbance at 450 nm. Three repetitions were conducted in triplicate.

### Clonogenic assay

Stable transfected cells (200 cells/well) were seeded into six-well plates and grown for 10 days. After fixed by 4% paraformaldehyde, the colonies were incubation with 0.1% crystal violet for 20 min. Then images were acquired and the number of colonies was counted.

### Flow cytometry analysis

For cell cycle analysis, cells were harvested and fixed with 70% ethanol, followed by sequential treatment with RNase A (100 μg/mL) and propidium iodide (PI) staining buffer. To analyze cell apoptosis, HUVECs treated as indicated were harvested and labeled with Annexin V-FITC and PI. Cell cycle distribution and apoptosis index were assessed by flow cytometry.

### Senescence-associated β-galactosidase (SA-β-gal) staining

A SA-β-gal staining kit (Beyotime) was used to evaluate cellular senescence via detection of β-galactosidase activity following the manufacturer’s protocols. Cells were plated in six-well plates and cultivated for 48 h. Then cells were washed with PBS and treated with fixative buffer for 15 min. After washed with PBS for three times, cells were incubated with premixed staining solution in a CO_2_-free atmosphere overnight. The images were acquired and SA-β-gal positive cells were counted under a microscope.

### Wound healing assay

Cells seeded on six-well plates were cultivated to nearly full confluence. A yellow pipette tip was used to scratch a wound on the monolayer cells. Then photographs of scratched cells were taken under a microscope to monitor wound closure at indicated time.

### Transwell assay

Transwell assays were conducted to evaluate the capacities of migration and invasion. Cells were resuspended in medium without FBS and counted. The 8-μm-pore chambers (Corning) were coated with (for invasion assay) or without (for migration assay) Matrigel (BD Biosciences) and inserted into 24-well plates. Resuspended cells were placed into upper chamber for transwell migration (2 × 10^4^ cells/well) and invasion (1 × 10^5^ cells/well) assay. Chambers were then incubated with medium containing 10% FBS in 24-well plates for 24 h. Penetrated cells were stained with crystal violet and images were acquired for cell count under a microscope.

### Conditioned medium (CM) collection

Stable transfected cells (ACHN-shNC, ACHN-sh#1) were re-suspended with DMEM/F-12 containing 10% FBS and plated in 60 mm dishes (5 × 10^5^ cells/dish). After incubation for 12 h, cells were washed and normal growth medium was replaced with serum-free medium (SFM) (5 ml/dish). After incubation for 24 h, cell culture supernatants from ACHN-shNC and ACHN-sh#1 cells were harvested. Cell debris in the supernatants was removed with a centrifuge.

### Enzyme-linked immunosorbent assay (ELISA)

The levels of VEGFA in conditioned medium from ACHN-shNC and ACHN-sh#1 cells were detected by using an ELISA kit (Sino Biological) according to the manufacturer’s protocols. Then a microplate reader (Bio-Rad) was used to measure absorbance at 450 nm.

### HUVECs recruitment assay

The Boyden chamber (Millipore) assay was performed to determine the effect of ccRCC cells on HUVECs recruitment. Briefly, HUVECs were resuspended with SFM and placed into upper chamber (2 × 10^4^ cells/well). Chambers were then incubated with indicated CMs (SFM, shNC-CM, sh#1-CM) or co-cultured with ccRCC cells (ACHN-shNC, ACHN-sh#1) in lower chamber for 24 h. Penetrated cells were stained with crystal violet and images were acquired for cell count under a microscope.

### Tube formation assay

Matrigel (BD Biosciences) (50 μl/well) was used to coat the wells of 96-well plates. After solidification for 1 h at 37 °C, HUVECs (4 × 10^4^ cells/well) were re-suspended with indicated CMs (SFM, shNC-CM, sh#1-CM) and plated onto the matrigel. After 6-h incubation at 37 °C for tube formation, tubules were observed and pictures were acquired by microscopy. ImageJ software was used to analyze the formed tubes.

### Western blotting

Immunoblotting assay was carried out as we previously described [16]. Cells were lysed using RIPA buffer containing protease and phosphatase inhibitors. The protein sample was resolved by SDS-PAGE, followed by transference of proteins onto PVDF membranes. The membrane was then treated with 5% bovine serum albumin, followed by overnight incubation with specific antibodies against Akt (1:1000, Cell Signaling Technology, CST), p-Akt (1:1000, CST), GSK-3β (1:1000, CST), p-GSK-3β (1:1000, CST), VEGFA (1:500, Sangon Biotech), SALL4 (1:1000, CST) and GAPDH (1:2000, Sangon Biotech). The corresponding HRP-conjugated secondary antibody and ECL substrate were used to visualize the protein bands.

### Immunofluorescence staining

Cells were plated and grown on coverslips overnight. After fixed in 4% paraformaldehyde, cells were treated with 0.5% triton X-100 for cell permeabilization. The coverslips were then immersed in blocking solution (Beyotime) for 1 h, followed by incubation with anti-p-GSK-3β (1:300, CST) and anti-p-Akt (1:200, CST) overnight. The Alexa Fluor 594-conjugated secondary antibody (1:200, Zsbio) was used for fluorescence labeling. Coverslips were incubated with DAPI for nuclei staining. Cells were observed and pictures were acquired by fluorescence microscope.

### In vivo tumorigenicity assay

Immunodeficient BALB/c mice (six-week-old) were provided by Experimental Animal Center of Fourth Military Medical University (FMMU) and kept under SPF conditions. The procedures involving animals received approval of the Animal Research Ethics Committee of FMMU and the research was conducted in accordance with institutional guidelines. For tumor growth assay, 786-O sublines (1 × 10^7^ cells) were implanted subcutaneously into the flanks of mice (six per group). A vernier caliper was used to measure tumor size every 5 days. The formula (volume = length × width^2^/2) was used to calculate tumor volume. Mice were sacrificed 5 weeks after inoculation. Tumor nodules were collected for further examination.

### Human tissue samples

Patient specimens (tumor and matched normal tissues) were acquired from ccRCC patients (*n* = 10) undergoing nephrectomy at Tangdu Hospital of FMMU. The patients received clinical and pathological diagnosis. Written informed consent was provided by the patients. This research gained the approval of Medical Ethics Committee of Tangdu Hospital.

### Immunohistochemistry (IHC) staining

Immunohistochemistry staining was carried out as we previously described [16]. Morphology analysis of formalin-fixed and paraffin-embedded (FFPE) sections were performed with hematoxylin and eosin staining. For IHC staining, heat-based antigen unmasking was conducted using microwave in citrate buffer after dewax and rehydration of sections. Tissue sections were exposed to 3% H_2_O_2_ to block endogenous peroxidase, followed by treatment with blocking solution for nonspecific binding. Subsequently the sections were stained with specific antibodies against SALL4 (1:100, Abcam) overnight. After incubation with corresponding secondary antibodies, a DAB substrate kit was used to visualize positive antigen binding. Haematoxylin counterstaining was performed and pictures were captured by microscopy.

### Bioinformatics analysis

To identify the expression pattern and clinical characteristics of SALL4, public datasets (TCGA Renal 2 and TCGA-KIRC cohorts) were analyzed in Oncomine (http://www.oncomine.org/) and UALCAN [17] (http://ualcan.path.uab.edu/) databases. The correlation between SALL4 expression and survival in ccRCC patients was assessed by GEPIA (http://gepia.cancer-pku.cn/) and Kaplan-Meier Plotter (http://kmplot.com/analysis/) databases. Genome-wide association analysis of SALL4 was performed with Cancer Regulome Tools (http://explorer.cancerregulome.org/). Gene correlation analysis, mutation analysis and methylation analysis were carried out using LinkedOmics [18] (http://www.linkedomics.org/) and TCGAportal (http://tumorsurvival.org/) databases.

### Statistical analysis

Group differences were determined using IBM SPSS 18.0 software. Experimental values are represented as means ± SD. Two-tailed Student’s *t*-test (two groups) and one-way ANOVA test (three or more groups) were conducted as appropriate for differences comparison. Kaplan-Meier method was employed to analyze patient survival and Pearson correlation coefficient was applied for gene expression correlation analysis. Difference was considered significant as indicated (* *P* < 0.05, ** *P* < 0.01, *** *P* < 0.001 and **** *P* < 0.0001).

## Results

### Upregulated SALL4 in ccRCC promotes tumor progression and indicates poor prognosis

**Fig. 1:**
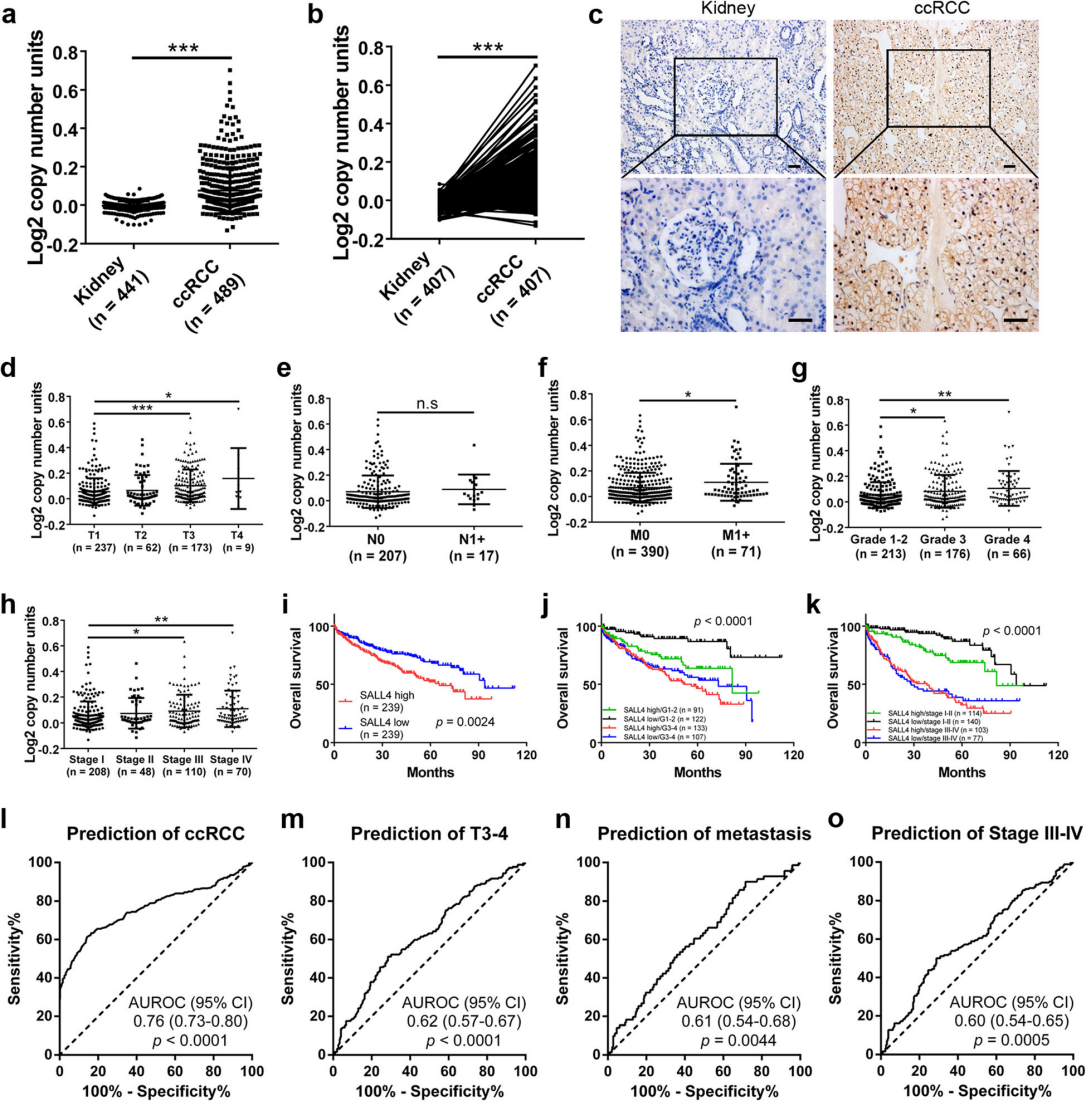
Upregulated SALL4 in ccRCC promotes tumor progression and indicates poor prognosis. **a** Relative SALL4 expression in normal kidney and ccRCC tissues. **b** Differential expression of SALL4 in 407 paired tissues from ccRCC patients. Data (**a**, **b**) were acquired from TCGA Renal 2 dataset via Oncomine database. **c** Representative IHC images of SALL4 in normal kidney and ccRCC tissues (scale bar, 20 μm). **d**-**h** High expression of SALL4 was positively correlated with tumor progression. Varied expression of SALL4 depending on tumor invasion (**d**), lymphatic metastasis (**e**), distant metastasis (**f**), histological grade (**g**) and AJCC stage (**h**). **i**-**k** Kaplan-Meier analysis of overall survival in ccRCC patients based on SALL4 expression. **l**-**o** Area under ROC curve (AUROC) analyses were performed to determine the diagnostic potential for indicated ccRCC patients. Data were acquired from TCGA Renal 2 dataset via Oncomine database. * *P* < 0.05, ** *P* < 0.001 and *** *P* < 0.001

To elucidate SALL4 expression signatures in human malignancies, we conducted pan-cancer analysis of public TCGA datasets in UALCAN database. Our results showed that SALL4 was significantly upregulated in most of human tumors (Additional file 1: Figure S1a), including ccRCC, indicating an oncogenic role for SALL4 in tumorigenesis. Further analysis of publicly available datasets was performed to evaluate the expression levels of SALL4 in ccRCC and normal kidney tissues. We found that SALL4 was dramatically overexpressed in ccRCC tissues compared with normal kidney tissues (Fig. 1a, b and Additional file 1: Figure S1b, c). To validate the upregulated SALL4 expression in ccRCC patients, we performed IHC staining in ccRCC and adjacent noncancerous samples from the same patients and observed higher protein levels of SALL4 at both plasmolemma and nucleus in ccRCC specimens (Fig. 1c). Moreover, SALL4 expression varied depending on tumor invasion depth, lymph node status, distant metastasis, histological grade and AJCC stage (Fig. 1d-h and Additional file 1: Figure S1d-g).

In addition, the correlation between SALL4 expression and ccRCC patients’ clinicopathological characteristics was analyzed to investigate the clinical significance of SALL4 in ccRCC. Our results revealed that SALL4 expression positively correlated with T stage, tumor grade and AJCC stage (Table 1, *P* < 0.05). Kaplan–Meier survival analysis revealed the close relationship between SALL4 expression and the prognosis of ccRCC patients. The overall survival time was significantly shorter in ccRCC patients with high SALL4 expression than in those with low SALL4 expression (Fig. 1i-k and Additional file 2: Figure S2). Univariate and multivariate analyses were performed to identify risk factors of prognosis in ccRCC patients. SALL4 was found to be an independent prognostic factor of overall survival for ccRCC patients (Table 2, *P* < 0.05). To further explore the diagnostic potential of SALL4, the receiver-operating characteristic (ROC) curve analysis was performed by measuring the area under the curve (AUROC). Our data indicated that SALL4 level could distinguish ccRCC patients from healthy controls (Fig. 1l) and diagnose patients with deeper invasion (T3–4) (Fig. 1m), distant metastasis (Fig. 1n) and advanced stage (Stage III-IV) (Fig. 1o). Taken together, these data suggest that upregulation of SALL4 contributes to ccRCC progression and is associated with poor prognosis of ccRCC patients.

**Table 1:** Association of SALL4 expression with clinicopathological parameters of ccRCC patients (TCGA Renal 2)

Parameters	Frequency (%)	SALL4 expression		*P* value
		< median	≥median	
Age (*n* = 481)				
< 60	222 (46.2)	112	110	0.822
≥ 60	259 (53.8)	128	131	
Sex (*n* = 481)				
Male	313 (65.1)	148	165	0.118
Female	168 (34.9)	92	76	
T stage (*n* = 481)				
T1	237 (49.3)	133	104	0.003
T2	62 (12.9)	35	27	
T3-T4	182 (37.8)	73	109	
N stage (*n* = 224)				
N0	207 (92.4)	109	98	0.065
N1+	17 (7.6)	5	12	
M stage(*n* = 461)				
M0	390 (84.6)	203	187	0.051
M1+	71 (15.4)	28	43	
Tumor Grade (*n* = 455)				
Grade 1–2	213 (2.0)	122	91	0.003
Grade 3	176 (38.7)	86	90	
Grade 4	66 (14.5)	22	44	
AJCC stage (*n* = 436)				
Stage I	208 (47.7)	116	92	0.047
Stage II	48 (11.0)	24	24	
Stage III	110 (25.2)	51	59	
Stage IV	70 (16.1)	26	44

**Table 2:** Cox regression analysis of prognostic factors associated with overall survival of ccRCC patients (*n* = 200)

Parameters	Univariate analysis			Multivariate analysis		
	HR	95% CI	*P* value	HR	95% CI	*P* value
Age						
< 60	1.354	0.846–2.168	0.207	–	–	–
≥ 60						
Sex						
Male	1.062	0.670–1.683	0.800	–	–	–
Female						
T stage						
T1-T2	3.529	2.217–5.618	< 0.001	2.377	0.945–5.983	0.066
T3-T4						
N stage						
N0	2.149	1.071–4.312	0.031	0.726	0.332–1.589	0.423
N1+						
M stage						
M0	5.290	3.291–8.502	< 0.001	4.375	2.381–8.038	< 0.001
M1+						
Tumor Grade						
Grade 1–2	2.451	1.485–4.047	< 0.001	1.190	0.674–2.102	0.548
Grade 3–4						
AJCC stage						
Stage I-II	3.776	2.326–6.128	< 0.001	0.967	0.355–2.639	0.948
Stage III-IV						
SALL4 level						
Low	2.058	1.289–3.285	0.003	1.844	1.129–3.012	0.015
High						

### SALL4 promotes ccRCC cells growth in vitro and in vivo

**Fig. 2:**
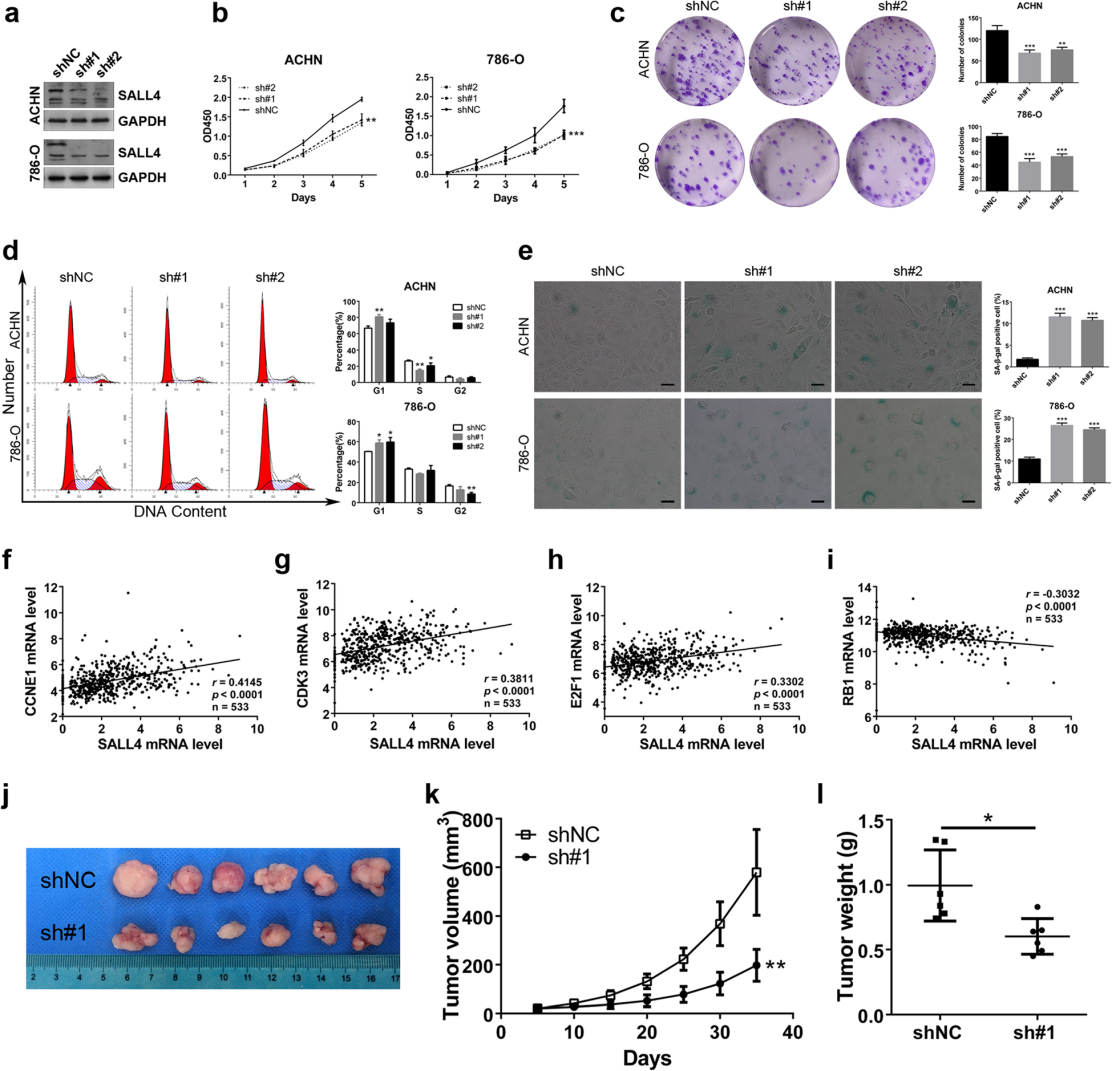
SALL4 promotes ccRCC cells growth in vitro and in vivo. **a** Western blot analyses of SALL4 expression in ccRCC cells stably expressing indicated shRNA (shNC, negative control shRNA; sh#1 and sh#2, shRNAs targeting SALL4). **b** The CCK-8 assays were performed in ACHN and 786-O cells treated with indicated shRNA. **c** Colony formation assays in ACHN and 786-O cells with indicated shRNA treatment. **d** Cell cycle distribution was examined by flow cytometry in ACHN and 786-O cells treated as indicated. **e** Cellular senescence was detected by SA-β-gal staining in ACHN and 786-O cells treated with shNC and shSALL4 (scale bar, 50 μm). **f**-**i** Scatter plot analyses were performed to determine the correlation between SALL4 and CCNE1 (**f**), CDK3 (**g**), E2F1 (**h**) and RB1 (**i**) mRNA expression levels in 533 ccRCC patients from TCGA database. Data were analyzed via LinkedOmics bioinformatics. **j** The image of dissected tumors from nude mice. **k**, **l** The growth curve (**k**) and their weights (**l**) of subcutaneous tumors formed by 786-O cells with indicated treatment. * *P* < 0.05, ** *P* < 0.001 and *** *P* < 0.001

To further validate its oncogenic activities in ccRCC, we set out to check the putative function of SALL4 on ccRCC cell growth. Lentiviral shRNA-mediated knockdown of SALL4 was conducted and downregulated SALL4 protein levels in ccRCC cells (ACHN, 786-O) were detected (Fig. 2a). The cells were then subjected to CCK-8 and colony formation assays to determine the influence of SALL4 downregulation on ccRCC cell proliferation. We found that knockdown of SALL4 in ACHN and 786-O cells resulted in slower growth rate compared with control cells (Fig. 2b). Similarly, the number of colonies formed by cells with downregulated SALL4 was significantly reduced (Fig. 2c). To test whether SALL4 also drives cell cycle progression, flow cytometry analysis was performed. The results showed that remarkable changes of cell cycle distribution were induced by SALL4 silencing in ccRCC cells. As indicated by increased G1-phase cells and decreased S/G2-phase cells, downregulation of SALL4 in ccRCC cells arrested cell cycle by restraining G1-S transition (Fig. 2d). Resistance to senescence or apoptosis has been identified as a hallmark of cancer cells and plays a crucial role in cell survival and tumorigenesis [19]. In particular, it has been demonstrated that some cells are more prone to senescence rather than apoptosis even following intensive exogenous stress [20]. SA-β-gal is the most frequently used marker for senescence and senescent cell exhibits high SA-β-gal activity. To further elucidate the functional role of SALL4 in cell senescence, ccRCC cells with stable SALL4-targeted or control shRNA were assayed using SA-β-gal staining kit. We observed that depletion of SALL4 in ACHN and 786-O cells upregulated SA-β-gal synthesis (Fig. 2e) indicating that SALL4 depletion triggered cells senescence. By analyzing a public dataset of 533 ccRCC patients from TCGA, we found that SALL4 mRNA level was significantly correlated with the transcripts of genes related to proliferation, senescence and cell cycle, including CCNE1 (*r* = 0.4145, *P* < 0.0001), CDK3 (*r* = 0.3811, *P* < 0.0001), E2F1 (*r* = 0.3302, *P* < 0.0001) and RB1 (*r* = − 0.3032, *P* < 0.0001) (Fig. 2f-i and Additional file 3: Figure S3, Additional file 4: Table S1). Next, to investigate the oncogenic activity of SALL4 in ccRCC tumorigenesis in vivo, tumor formation was evaluated by subcutaneous inoculation of 786-O sublines in nude mice. we found that downregulation of SALL4 in ccRCC cells resulted in a dramatic decrease in tumorigenic potential, as evidenced by decreased tumor size, repressed tumor growth and reduced tumor weight (Fig. 2j-l). Together, these findings validate that SALL4 drives ccRCC cell growth by promoting cell cycle progression and restraining cell senescence.

### SALL4 promotes ccRCC cells migration and invasion in vitro

**Fig. 3:**
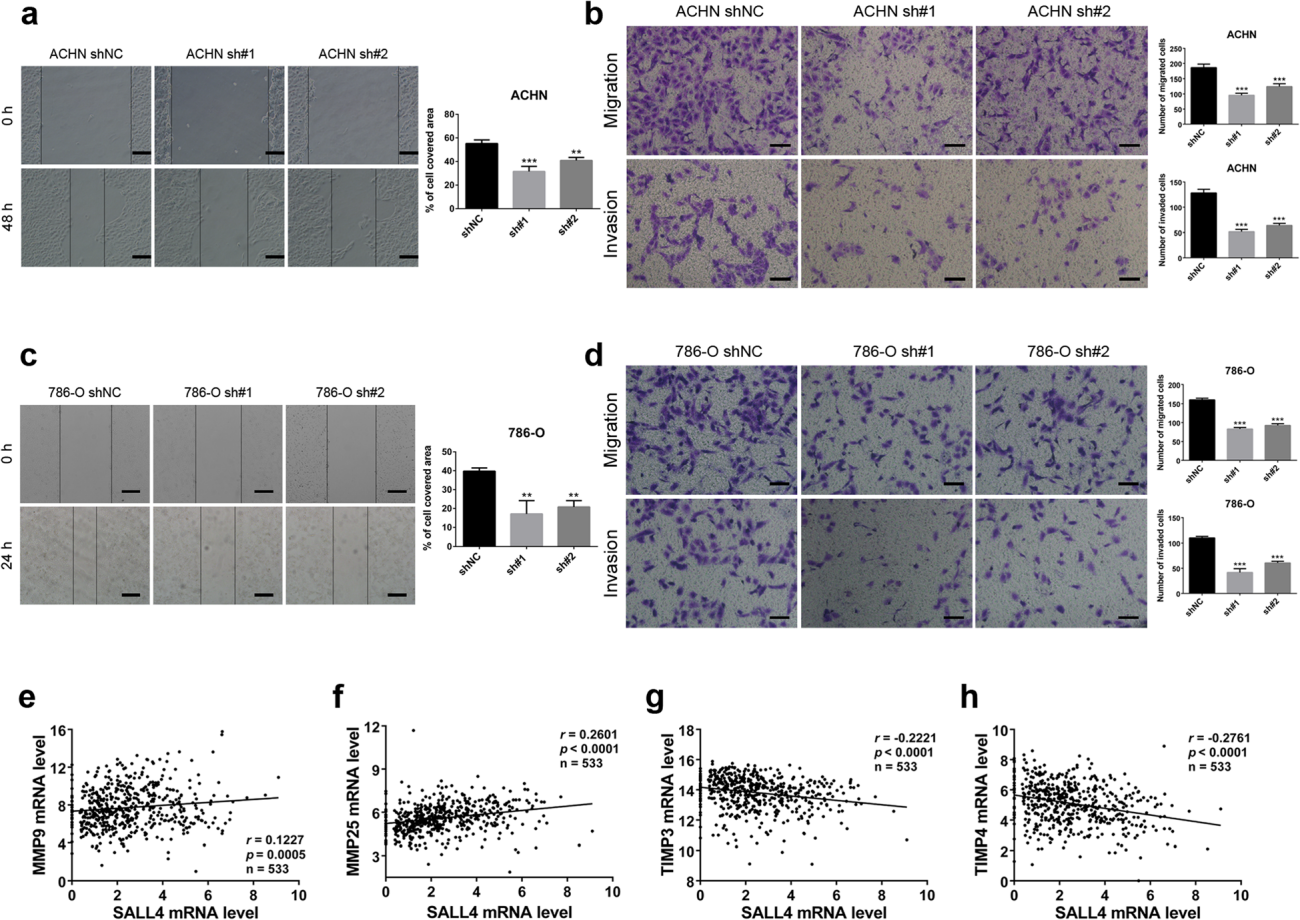
SALL4 promotes ccRCC cells migration and invasion in vitro. **a**, **c** Cell migration ability was evaluated by wound healing assays in ACHN (**a**) and 786-O (**c**) cells with indicated shRNA treatment (scale bar, 200 μm). **b**, **d** Transwell migration and matrigel invasion assays were conducted to determine the cell migration and invasion capabilities in ACHN (**b**) and 786-O (**d**) cells treated as indicated (scale bar, 100 μm). **e**-**h** Scatter plot analyses were performed to determine the correlation between SALL4 and MMP9 (**e**), MMP25 (**f**), TIMP3 (**g**) and TIMP4 (**h**) mRNA expression levels in 533 ccRCC patients from TCGA database. Data were analyzed via LinkedOmics bioinformatics. ** *P* < 0.001 and *** *P* < 0.001

Next, to explore whether SALL4 also function as a prometastatic factor in ccRCC, we performed a series of loss-of-function studies in ACHN and 786-O cells stably transfected with SALL4-targeted or control shRNA. The wound healing assays demonstrated that SALL4 downregulation markedly suppressed cell migration to delay healing of the scratched cell monolayer in ccRCC cells (Fig. 3a, c). Similar results were observed in transwell migration assays. We found that SALL4 silencing in ccRCC cells significantly impaired the migratory ability as measured by cells attached to the lower membrane surfaces. Consistently, in matrigel invasion assays of ACHN and 786-O cells, less cells were observed to penetrate through the matrigel barrier upon SALL4 knockdown, indicating a decrease in invasion potential (Fig. 3b, d). These results were consistent with our finding that SALL4 was upregulated in metastatic ccRCC tumors (Fig. 1f). The epithelial-mesenchymal transition has been reported to be involved in SALL4-mediated tumor metastasis [21]. In agreement with previous findings, we found that compared with the control cells, SALL4-deficient ACHN cells seemed to exhibit a tighter organization of cells in colonies (Additional file 5: Figure S4a). In addition, we analyzed the RNA-seq data of 533 ccRCC patients from TCGA and found that SALL4 mRNA level was significantly correlated with the transcripts of matrix metalloproteinases (MMPs) and tissue inhibitors of metalloproteinases (TIMPs) related to tissue remodeling and tumor invasiveness [22, 23], including MMP9 (*r* = 0.1227, *P* = 0.0005), MMP25 (*r* = 0.2601, *P* < 0.0001), TIMP3 (*r* = − 0.2221, *P* < 0.0001) and TIMP4 (*r* = − 0.2761, *P* < 0.0001) (Fig. 3e-h and Additional file 6: Figure S5, Additional file 7: Table S2). Collectively, these results support that SALL4 functions as a prometastasis oncogene in ccRCC progression.

### SALL4 expression is associated with endothelium development and vasculogenesis

**Fig. 4:**
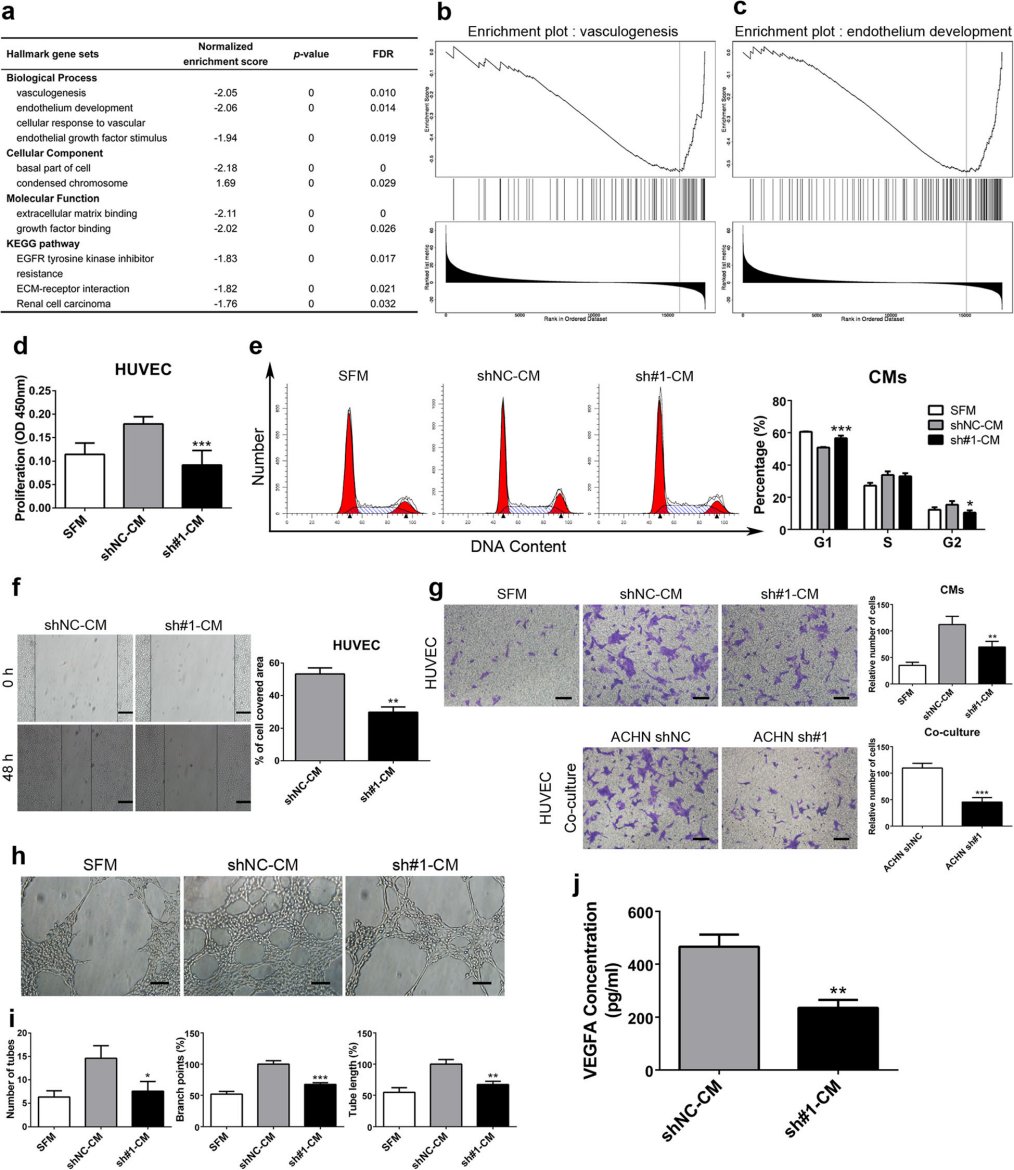
SALL4 induces ccRCC angiogenesis in vitro. **a** Enriched gene ontology (GO) term and KEGG pathway analysis of genes significantly correlated with SALL4 in ccRCC. **b**, **c** Gene set enrichment analysis (GSEA) profiles for the genes significantly correlated with SALL4 in ccRCC. Data (**a**-**c**) from TCGA database were analyzed via LinkedOmics bioinformatics. **d** HUVECs were treated with serum-free medium (SFM) or conditioned medium (CMs) from ACHN-shNC/shSALL4 cells. After treatment for 72 h, CCK-8 assays were performed to evaluate cell proliferation. **e** Flow cytometry analyses of cell cycle distribution in HUVECs treated with SFM or CMs from ACHN-shNC/shSALL4 cells. **f** Wound-healing assay was performed to determine the migration of HUVECs with CMs treatment (scale bar, 200 μm). **g** Representative images and quantification analyses of the recruitment of HUVECs co-cultured with indicated CMs (*upper*) or ACHN-shNC/shSALL4 cells (*lower*) in lower chambers (scale bar, 100 μm). **h, i** Representative images (**h**) and quantification analyses (**i**) of tube formation in HUVECs treated with SFM or indicated CMs (scale bar, 100 μm). **j** The expression level of VEGFA in CMs from ACHN-shNC/shSALL4 cells was detected by ELISA assay. * *P* < 0.05, ** *P* < 0.001 and *** *P* < 0.001

To further characterize SALL4’s biologic functions and discern its oncogenic mechanisms, we first characterized the differential association signature between SALL4 mRNA level and individual gene expression by analyzing the RNA-seq data of 533 ccRCC patients from TCGA. As shown in Additional file 8: Figure S6a, we identified 6315 (dark red dots) and 2862 (dark green dots) genes that were positively and negatively correlated with SALL4 (FDR < 0.01), indicating that SALL4 may exert a widespread influence on the transcription profiles. We then performed cluster analysis of the correlation profiles and constructed a heatmap to visualize the top 50 most significant genes that were positively and negatively correlated with SALL4 transcripts (Additional file 8: Figure S6b, c). Statistical scatter plots for top-ranked genes were created and revealed a strong positive correlation between SALL4 mRNA level and NOD-like receptor family CARD domain containing 5 (NLRC5) gene expression (positive rank #2, *r* = 0.62, *P* = 6.66e-57) (Additional file 8: Figure S6d). NLRC5 was previously reported to be upregulated in ccRCC and positively associated with tumor progression [24]. Recent evidence indicates that NLRC5 is implicated in vascular remodeling and required for vascular intimal hyperplasia [25]. we next conducted gene ontology term enrichment and KEGG pathway analyses of SALL4-associated genes and found that SALL4 expression significantly correlated with genes controlling vasculogenesis, endothelium development and cellular response to vascular endothelial growth factor stimulus (Fig. 4a), confirming the potential role of SALL4 in modulating vascular remodeling. Vasculogenesis refers to the formation of new blood vessels. Endothelium development is a complex process in which endothelial cells are oriented towards their specific fate and specialized endothelial cells form the endothelium of an organ such as vasculature, lymph vessel and heart. Furthermore, gene set enrichment analysis (GSEA) were performed in human ccRCC samples and revealed a large number of significantly enriched gene sets, including vasculogenesis (Fig. 4b) and endothelium development (Fig. 4c). Genome-wide association of SALL4 mRNA expression with multifarious molecular signatures, including gene expression, protein level (reverse phase protein arrays, RPPA), microRNA expression, DNA methylation, copy number variation and somatic mutation, was analyzed and visualized using a circos plot (Additional file 8: Figure S6e) and a network (Additional file 8: Figure S6f). In agreement with our observations in Additional file 8: Figure S6a-d, we found that the transcription levels of NLRC5 and SALL4 mRNA expression were strongly associated in ccRCC patients (Additional file 8: Figure S6f). In summary, these data suggest that SALL4 may be involved in vasculogenesis and vascular remodeling.

### SALL4 induces ccRCC angiogenesis in vitro

Given the potential role of SALL4 in vasculogenesis and vascular remodeling, we sought to investigate whether SALL4 modulates angiogenesis in ccRCC. In response to treatment with CM collected from ACHN sublines, we observed that HUVECs cultured in CM from ACHN-sh#1 cells showed significant reduction in viable cell number as measured by CCK-8 assay (Fig. 4d). Flow cytometry analysis of HUVECs treated with CM from ACHN-sh#1 cells revealed an arrested cell cycle compared with that of cells treated with CM from ACHN-shNC cells (Fig. 4e), but no significant alteration in cell apoptosis was observed (Additional file 5: Figure S4b, c). Scratch assay was performed on HUVECs treated with CMs. We found that migration of HUVECs was markedly alleviated after treatment with CM from ACHN-sh#1 cells (Fig. 4f). In HUVECs recruitment assay, we observed that the chemotactic response of HUVECs migrating toward CM from ACHN-sh#1 cells was drastically suppressed in comparison with that of cells stimulated with CM from ACHN-shNC cells (Fig. 4g). To explore the direct effect of SALL4 deficiency on the function of endothelial cells, we established a coculture system of HUVECs with ACHN sublines. Consistent with the above findings, we noticed that much fewer HUVECs were recruited toward ACHN cells lacking SALL4 (Fig. 4g). To substantiate the relevance of SALL4 to angiogenesis in ccRCC, HUVECs were treated with CMs from ACHN sublines and assayed for tube formation. We observed that as expected, HUVECs exhibited diminished tube formation activity following stimulation with CM from SALL4-deficient ACHN cells (Fig. 4h, i). Furthermore, we analyzed the levels of VEGFA in CMs from ACHN sublines and found that the concentration of VEGFA in CM from ACHN-sh#1 cells decreased (Fig. 4j). Taken together, these in vitro observations validate the potent role of SALL4 in ccRCC angiogenesis.

### SALL4 downregulation attenuates the activation of Akt/GSK-3β signaling and VEGFA expression in ccRCC

**Fig. 5:**
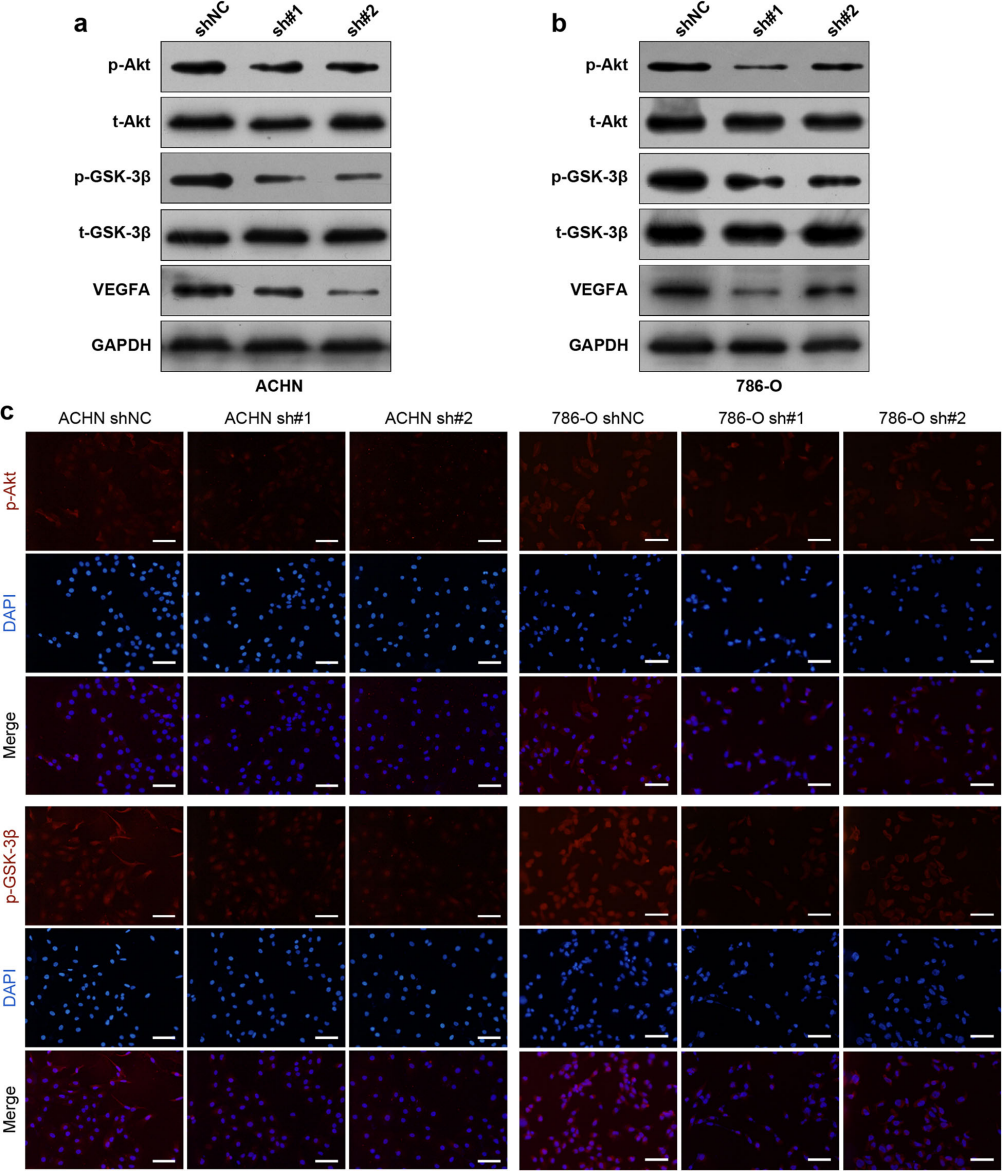
SALL4 downregulation attenuates the activation of Akt/GSK-3β signaling and VEGFA expression in ccRCC. **a**, **b** Western blot analyses for protein levels of total Akt (t-Akt), phosphorylated Akt (p-Akt), total GSK-3β (t-GSK-3β), phosphorylated GSK-3β (p-GSK-3β) and VEGFA in ACHN (**a**) and 786-O (**b**) cells with indicated treatment. **c** Immunofluorescence staining of p-Akt and p-GSK-3β in ACHN (*left*) and 786-O (*right*) cells with indicated treatment (scale bar, 50 μm)

SALL4 was previously implicated in chromatin remodeling via modulation recruitment of nucleosome remodeling deacetylase (NuRD) complex [26]. The tumor suppressor PTEN has been reported to be a target gene repressed by SALL4 and counteract the PI3K pathway [13]. To validate the involvement of PI3K/Akt signaling in SALL4-mediated oncogenic activities in ccRCC, we evaluated the effects of SALL4 downregulation on PI3K pathway markers. Immunoblotting analysis of lysates from ccRCC cells revealed that SALL4 knockdown decreased the levels of phosphoprotein markers of PI3K pathway activation (p-Akt, p-GSK3β) without altering the total protein levels in ACHN and 786-O cells (Fig. 5a, b). Consistently, immunofluorescent staining (Fig. 5c) further confirmed the observations in western blot assay. The extensive crosstalk and a positive feedback loop have been reported to exist between the PI3K/Akt and VHL/HIF pathways [7], contributing to ccRCC tumorigenesis and progression. Given the functional impact of SALL4 on angiogenesis and decreased VEGFA level in CM from SALL4-downregulated ACHN cells, we further investigated whether SALL4 modulates the synthesis of VEGFA, a downstream target of VHL/HIF pathway. We observed that depletion of SALL4 led to a dramatic reduction in VEGFA protein levels in ACHN and 786-O cells (Fig. 5a, b), thus suggesting a link between SALL4 expression and VHL/HIF pathway. Collectively, these results indicate that SALL4 expression positively correlates with the activation of Akt/GSK-3β signaling and VEGFA expression.

### Mutation of VHL is involved in the upregulation of SALL4 in ccRCC

**Fig. 6:**
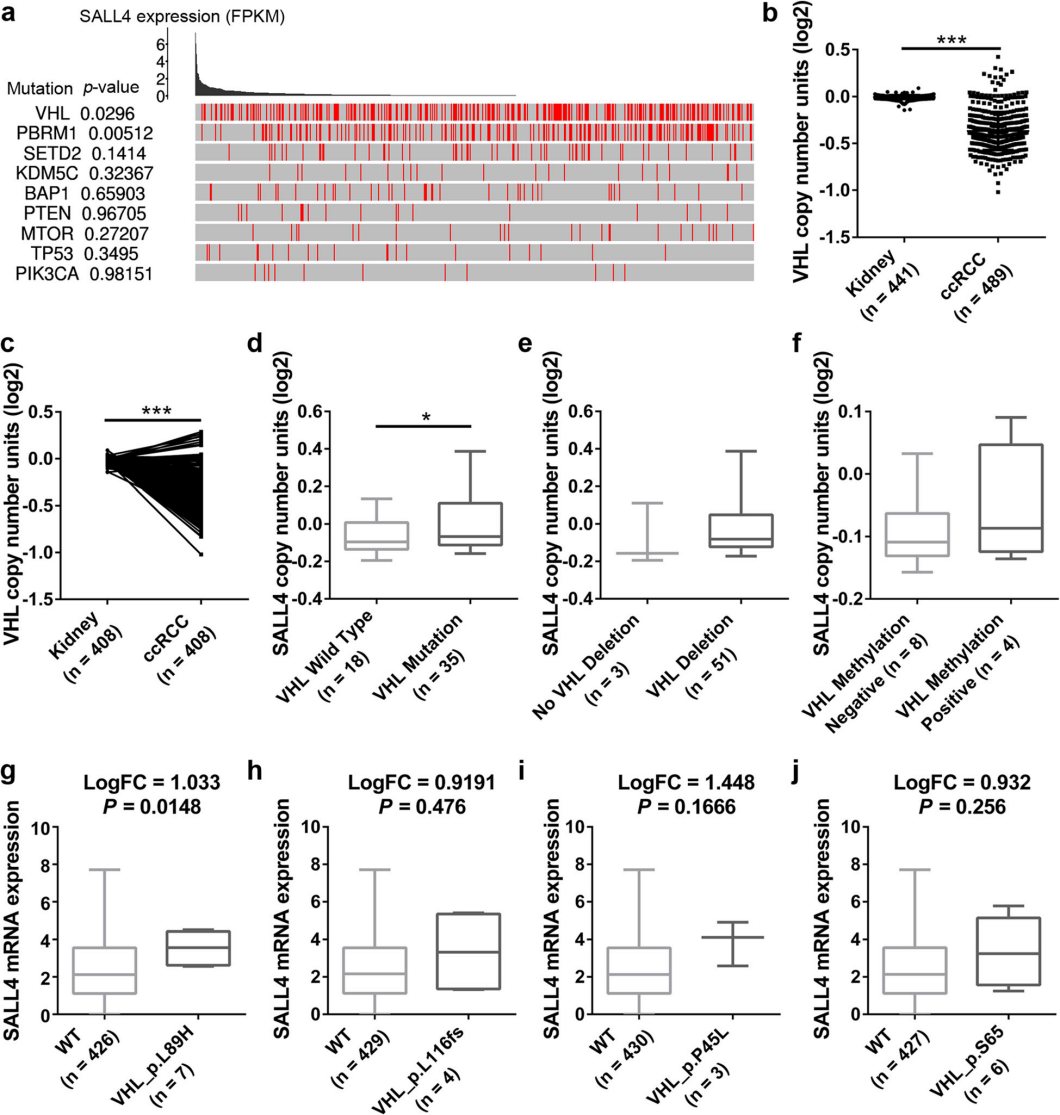
Mutation of VHL is involved in the upregulation of SALL4 in ccRCC. **a** The permutation test analysis of SALL4 expression between driver mutated (red) and non-mutated (gray) ccRCC samples from TCGA database. **b** Relative VHL expression in normal kidney and ccRCC tissues. **c** Differential expression of VHL in 408 paired tissues from ccRCC patients. Data (**b**, **c**) were acquired from TCGA Renal 2 dataset via Oncomine database. **d**-**f** Analysis of SALL4 expression in association with VHL mutation (**d**), deletion (**e**) and methylation (**f**) status in ccRCC patients from Beroukhim Renal 2 dataset via Oncomine database. **g**-**j** Functional impact of VHL site mutations on SALL4 gene expression in ccRCC patients from TCGA database. Data were analyzed via LinkedOmics bioinformatics. FC, fold change; WT, wild type

VHL protein has emerged as a potent tumor suppressor governing various signaling pathways that promote oncogenic phenotypes and fitness. Mutations in VHL are observed in roughly 70–80% of ccRCC and identified as causal event for ccRCC initiation and progression [27]. To investigate whether SALL4 overexpression is enriched in tumors with driver gene mutations, we analyzed the association between SALL4 expression and the mutation profile of most commonly altered genes (VHL, PBRM1, SETD2, KDM5C, BAP1, PTEN, MTOR, TP53 and PIK3CA) in ccRCC. Our results revealed a significant difference of SALL4 expression between tumors with VHL or PBRM1 mutations and non-mutated cases (Fig. 6a). Additionally, we explored the expression of VHL in TCGA Renal 2 dataset via Oncomine database and observed that VHL expression was significantly decreased in ccRCC samples when compared with that in normal kidney tissues (Fig. 6b, c). We then evaluated the relationship between SALL4 gene expression and the genetic status of VHL in ccRCC patients. Our data indicated that SALL4 gene copy number was higher in tumors with VHL deletion and methylation, especially VHL mutation (Fig. 6d-f), suggesting that SALL4 amplification was significantly co-occurred with VHL lesions. Nevertheless, no significant difference of SALL4 expression was observed among ccRCC patients with different VHL mutation subtypes (Additional file 9: Figure S7a). Next, to further acquire a better understanding of the functional role of VHL mutation in SALL4 expression, we analyzed SALL4 mRNA levels in ccRCC patients with or without different VHL point mutations (Fig. 6g-j and Additional file 9: Figure S7b-k). As shown in Fig. 6 g, a significant positive association was observed between VHL_p.L89H point mutation and SALL4 gene expression (*P* < 0.05).

Prior evidence demonstrates that VHL deficiency drives genome-wide changes in DNA methylation which has been implicated in the pathogenesis of ccRCC [28]. In addition, it is well-established that DNA methylation alterations are highly associated with aberrant expression of genes. We thus infer that DNA methylation may be also involved in VHL mutation-mediated upregulation of SALL4. By analyzing the correlation between SALL4 methylation status and its mRNA expression (Additional file 10: Figure S8), we found that six methylation regions of SALL4 were markedly hypomethylated in ccRCC tumor samples in comparison with paired normal kidney samples. Among the six hypomethylated SALL4 regions, a negative correlation between methylation status of chr20:51800437 and chr20:51800155 and SALL4 mRNA expression was observed in both ccRCC tumor samples and paired normal kidney samples (Additional file 10: Figure S8). Further exploration are required to determine the exact prevalence of promoter hypomethylation in association with SALL4 upregulation. Together, these data suggest that VHL mutation can function as a potential mechanism for SALL4 upregulation.

## Discussion

Recent studies have implicated high expression levels of SALL4 in tumorigenesis of numerous human malignancies including acute myeloid leukemia [26], hepatocellular carcinoma [13], endometrial cancer [29] and esophageal squamous cell carcinoma [21]. In this report, we discovered that SALL4 is frequently overexpressed in ccRCC patients and positively correlated with tumor progression. Given the aberrant expression in human cancers, SALL4 has been identified as a cancer biomarker. In human acute myeloid leukemia, the expression of SALL4 is associated with cancer progression and predictive of treatment outcomes [30]. Enhanced SALL4 expression is observed in early-stage breast cancer tissues [31] and serum of hepatocellular carcinoma patients [14], suggesting a potential role for SALL4 in early cancer detection and diagnosis. Patients with higher SALL4 levels exhibit poorer overall survival in hepatocellular carcinoma [14] and endometrial cancer [29]. Consistently, our results indicated that SALL4 can function as an independent prognostic factor for ccRCC patients. The clinical significance of SALL4 in ccRCC patients and its important role in cancer genesis and progression will shed light on novel diagnosis and therapy for ccRCC patients in the future.

SALL4 has emerged as a transcription factor governing multiple biologic processes in the initiation and development of human cancers [32]. Previous studies have identified SALL4 as a tumor-promoting oncogene. Downregulation of SALL4 suppresses cell proliferation and induces cell cycle arrest in acute myeloid leukemia [30], breast cancer [31] and endometrial cancer [29]. In contrast, forced expression of SALL4 prominently promotes tumor growth and results in accelerated tumorigenesis in liver cancer [13], nasopharyngeal carcinoma [11] and cervical cancer [33]. Consistent with prior findings, we found that SALL4 substantially enhances ccRCC cell growth and tumorigenic potential both in vitro and in vivo. In addition, a significant increase in cell apoptosis is frequently observed in SALL4-deficient cells of acute myeloid leukemia [26], esophageal squamous cell carcinoma [21] and endometrial cancer [29], which may also account for the growth suppression mediated by SALL4 knockdown. Moreover, cellular senescence, characterized by telomere shortening, has been identified as a candidate anticancer mechanism that potently suppresses tumorigenesis by limiting proliferation of tumor cells [19, 34]. However, there is no report about the action of SALL4 on cell senescence. Our results, for the first time, demonstrated that SALL4 knockdown in ccRCC cells induces a significant senescence response in vitro. Even though SALL4 has been reported to be strongly associated with tumor metastasis and promote migration and invasion in various cancers [12, 29], its functional role in ccRCC metastasis remains elusive. Here, our present study revealed that a dramatic decrease in migratory and invasive ability was observed upon SALL4 downregulation, suggesting a prometastatic role for SALL4 in ccRCC. Further investigations have indicated that SALL4 can drive tumor metastasis via induction of epithelial-mesenchymal transition (EMT) or direct transcriptional upregulation of c-Myc [21, 33]. In this report, we found that SALL4 significantly correlates with the levels of MMPs and TIMPs which are related to tissue remodeling and tumor invasiveness, thus suggesting a potential mechanism for SALL4-mediated metastatic behavior without EMT involvement.

Despite the protumorigenic and prometastatic activities of SALL4 in tumor progression, our results unveiled a novel role for SALL4 in angiogenesis. The vasculature functions as an important energy-supplying system and dysregulation of angiogenesis is strongly associated with tumor progression [35]. The formation of tumor vasculature is indispensable for supporting tumor growth and metastatic dissemination of cancer cells [36]. Enhanced vascularization has been identified as a prominent feature of ccRCC. It has been reported that the VEGF level, endothelial cell proliferation fraction and vessel density are progressively increased in high-grade ccRCC [37]. These observations support that targeting tumor angiogenesis can be a new therapeutic strategy to halt cancer progression. Recent studies have demonstrated that targeted therapies, such as VEGF inhibitors, are capable of pruning tumor vessels and suppressing tumor growth, thus benefiting patients with metastatic ccRCC [38]. Given the oncogenic activities of SALL4 in ccRCC, we propose that SALL4 may also play an active role in angiogenesis. In this work, for the first time, we reported that SALL4 is involved in endothelium development and vasculogenesis. Furthermore, we provided evidence that SALL4 promotes angiogenesis in vitro. We found SALL4 downregulation in ccRCC cells attenuates the recruitment of endothelial cells. Treatment with CM from SALL4-deficient ccRCC cells induces endothelial cell dysfunction by alleviating cell proliferation, migration and tube formation in HUVECs.

It has been reported that SALL4 negatively regulates PTEN expression by forming a transcriptional repression complex with NuRD [13, 26]. PTEN is a well-established negative regulator of PI3K/Akt pathway. Accumulating evidence has demonstrated that the PI3K/Akt pathway is constitutively activated in multiple tumors and drives ccRCC initiation and progression [7]. Targeted therapies abrogating PI3K/Akt/mTOR pathway have exhibited initial anticancer activity in ccRCC [1]. In agreement with prior findings, our results showed that activation of Akt/GSK-3β signaling is involved in SALL4-mediated oncogenic behavior in ccRCC. A positive feedback loop between PI3K/Akt and VHL/HIF pathway has been implicated in ccRCC tumorigenesis [7]. VHL deficiency in ccRCC results in accumulating HIF and drives transcriptional activation of downstream target genes, such as VEGF that can function as an intermediary in activation of PI3K/Akt pathway [39]. The subsequent activation of mTORCs in turn contributes to HIF upregulation. These signaling pathways can cross talk with each other at several levels to fine-tune the signaling network. In this study, we found that SALL4 downregulation results in decreased levels of pro-angiogenic factor VEGFA in both ccRCC cells and conditioned medium. These findings support a notion that SALL4 can manipulate the synthesis and secretion of VEGFA, which may be responsible for the role of SALL4 in angiogenesis. Although we provide evidence that SALL4 can exert an effect on the activation of Akt/GSK-3β signaling and VEGFA expression, the underlying mechanism still remain to be determined.

Mutation of VHL tumor suppressor is frequently observed in ccRCC patients and identified as a causal event for tumor evolution [27]. In combination with genetic alterations in cell cycle-related genes, VHL loss act cooperatively to drive ccRCC initiation and progression [3, 40]. It has been reported that inactivation of VHL results in genome-wide enhancer and superenhancer remodeling and contributes to oncogenic transcription in human ccRCC [41]. Further investigation demonstrates that combined deletion of VHL, TP53 and RB1 induces ccRCC formation in mice and causes significant changes in global transcriptional profiles, as evidenced by upregulation of multitudinous genes that are important for HIF signaling, DNA replication and cell cycle progression [40]. Little is known about the impact of VHL mutation on SALL4 expression. Interestingly, our present study revealed a significant correlation between VHL mutation and SALL4 expression. Elevated SALL4 levels were observed in ccRCC patients with VHL mutation, deletion or methylation. Furthermore, our results indicated that VHL_p.L89H point mutation can impose significant influence on SALL4 gene expression. Based on these above findings, it is plausible that VHL mutation may, to some extent, account for the overexpression of SALL4 in ccRCC. Additionally, it is widely acknowledged that DNA methylation is associated with chromatin remodeling and involved in transcriptional regulation of gene expression [42, 43]. Previous studies have shown that aberrant hypomethylation in SALL4 promotor is strongly correlated with SALL4 upregulation in myelodysplastic syndromes and acute myeloid leukemia [44, 45]. The findings that VHL deficiency drives genome-wide changes in DNA methylation profile [28] may carry further implications for the upregulation of SALL4 in ccRCC. In this study, a significant association between SALL4 expression and DNA methylation was observed (Additional file 8: Figure S6e and Additional file 10: Figure S8). We found that DNA hypomethylation is enriched in SALL4 promoter in ccRCC patients. The exact prevalence of promoter hypomethylation in association with SALL4 upregulation remains to be illuminated by additional explorations. Even though our data suggest a potential role for VHL mutation in SALL4 overexpression, further investigations are necessary to elucidate the precise mechanisms for upregulated SALL4 in ccRCC.

## Conclusions

In summary (Additional file 11: Figure S9), we provide evidence that upregulated SALL4 expression is strongly correlated with tumor progression and poor prognosis in ccRCC. we establish that SALL4 functions as an oncoprotein by promoting ccRCC growth and metastasis. Moreover, our findings unveil a previously uncharacterized role for SALL4 in angiogenesis. We further show that Akt/GSK-3β signaling and VEGFA are involved in SALL4-mediated oncogenic activities. In addition, our observations implicate VHL mutation and DNA hypomethylation in SALL4 upregulation. Collectively, these findings demonstrate a potent role for SALL4 in ccRCC progression and establishe SALL4-targeted therapy as a promising strategy for ccRCC treatment.

## Supplementary information

**Additional file 1:Figure S1.** SALL4 is upregulated in ccRCC in the TCGA project. **a** Pan-cancer analysis for SALL4 expression in normal and tumor tissues. **b** Differential expression of SALL4 in normal kidney and ccRCC tissues. **c** Relative SALL4 expression in normal kidney and ccRCC subtypes. **d**-**g** Analysis of SALL4 expression based on lymphatic metastasis (**d**), AJCC stage (**e**, **f**) and histological grade (**g**). n.s = no significance, * *P* < 0.05, ** *P* < 0.001, *** *P* < 0.001 and **** *P* < 0.0001.

**Additional file 2:Figure S2.** Upregulation of SALL4 predicts poor prognosis in ccRCC patients from TCGA database. **a** Overall survival analysis for the overall survival of ccRCC patients via Kaplan-Meier plotter. **b** Kaplan-Meier analysis of overall survival in ccRCC patients based on SALL4 expression via GEPIA. **c**, **d** Kaplan-Meier survival curves for ccRCC patients based on SALL4 expression and histological grade by UALCAN bioinformatics. **e**, **f** Kaplan-Meier survival analyses for ccRCC patients depending on SALL4 expression and AJCC stage via TCGAportal.

**Additional file 3:Figure S3.** Pearson correlation analysis of SALL4 mRNA expression with the transcripts of proliferation-associated genes. Scatter plots depicting the significant correlation between SALL4 expression and the mRNA levels of CCNA2, CCNB1, CCND2, CDK1 (**a**), CDK2, CDKN1A, CDKN1B, CDKN1C (**b**), MKI67, PCNA, FOXM1, PLK1 (**c**), MYBL2, BUB1, TOP2A and E2F3 (**d**). Data were acquired from TCGA database and analyzed by LinkedOmics.

**Additional file 4:Table S1.** Correlation between SALL4 and proliferation-associated genes.

**Additional file 5:Figure S4.a** Morphological observation of ACHN cells with indicted shRNA treatment under light microscope (scale bar, 50 μm). **b** Flow cytometry analysis of apoptosis in HUVECs treated with indicted SFM or CMs. **c** Quantification analysis of cell apoptosis in HUVECs with indicted treatment.

**Additional file 6:Figure S5.** Pearson correlation analysis of SALL4 mRNA expression with the transcripts of matrix metalloproteinases (MMPs). Scatter plots depicting the significant correlation between SALL4 expression and the mRNA levels of MMP3, MMP12, MMP13 (**a**), MMP17, MMP19, MMP20 (**b**), MMP21, MMP23B and MMP26 (**c**). Data were acquired from TCGA database and analyzed by LinkedOmics.

**Additional file 7:Table S2.** Association of SALL4 with matrix metalloproteinases (MMPs) and tissue inhibitors of metalloproteinases (TIMPs).

**Additional file 8:Figure S6.** Integrative multi-omics analysis of SALL4 mRNA expression in ccRCC. **a** Volcano plot of Pearson correlation coefficient analysis of SALL4 with genes differentially expressed in ccRCC. Red (green) dots represent genes positively (negatively) correlated with SALL4. **b**, **c** The heat maps showing the top 50 genes exhibiting significant positive (**b**) and negative (**c**) correlation with SALL4 in ccRCC. **d** Pearson correlation of SALL4 gene expression with NLRC5 transcript in ccRCC patients. Data (**a**-**d**) from TCGA database were analyzed via LinkedOmics bioinformatics. **e** Genome-wide association of SALL4 mRNA expression with multifarious molecular features in ccRCC. The arcs connected pairs of dots representing the features to indicate statistically significant associations. **f** The significant correlation between SALL4 gene expression with multiple molecular features were visualized using network plot. Data (**e**, **f**) from TCGA database were analyzed using Regulome Explorer.

**Additional file 9:Figure S7.**. Functional impact of VHL mutation on SALL4 mRNA expression in ccRCC patients. **a** Analysis of SALL4 expression difference among ccRCC patients with indicated VHL mutation types. Data were acquired from TCGA database and analyzed by TCGAportal. **b**-**k** Association between SALL4 mRNA expression and VHL point mutations in ccRCC patients. Data were acquired from TCGA database and analyzed by LinkedOmics.

**Additional file 10:Figure S8.**. Association analysis of SALL4 gene expression with its probe methylation in primary ccRCC tumor and matched normal tissues. Data were analyzed by TCGAportal.

**Additional file 11:Figure S9.**. The schematic diagram for elevated SALL4-mediated tumorigenesis and angiogenesis in ccRCC. Ub, ubiquitin; EC, endothelial cell; mTORC, mTOR complex; HRE, hypoxia response element.

## Data Availability

All data involved in the conclusions of this study are available in this article and its additional files. The datasets used for bioinformatics analysis are downloaded from Oncomine (http://www.oncomine.org/) and UALCAN (http://ualcan.path.uab.edu/), Cancer Regulome Tools (http://explorer.cancerregulome.org/), Kaplan-Meier Plotter (http://kmplot.com/analysis/), GEPIA (http://gepia.cancer-pku.cn/), LinkedOmics (http://www.linkedomics.org/) and TCGAportal (http://tumorsurvival.org/).

## Abbreviations

AUROC: Area under the receiver-operating characteristic curve
CCK-8: Cell counting kit-8
CCNE1: Cyclin E1
ccRCC: Clear cell renal cell carcinoma
CDK3: Cyclin-dependent kinase 3
CM: Conditioned medium
HIF: Hypoxia-inducible factor
HUVEC: Human umbilical vein endothelial cell
IHC: Immunohistochemistry
MMP: Matrix metalloproteinase
NLRC5: NOD-like receptor family CARD domain containing 5
NuRD: Nucleosome remodeling deacetylase
PI: Propidium iodide
RPPA: Reverse phase protein arrays
SA-β-gal: Senescence-associated β-galactosidase
SALL4: Sal-like 4
SFM: Serum-free medium
TIMP: Tissue inhibitors of metalloproteinase
VEGF: Vascular endothelial growth factor
VHL: Von Hippel-Lindau
WT: Wild type
