# Supplementary figures and images for: VHL mutation-mediated SALL4 overexpression promotes tumorigenesis and vascularization of clear cell renal cell carcinoma via Akt/GSK-3β signaling
Source: J Exp Clin Cancer Res. 2020 Jun 8;39:104. doi: 10.1186/s13046-020-01609-8 (PMC7278163; doi:10.1186/s13046-020-01609-8)

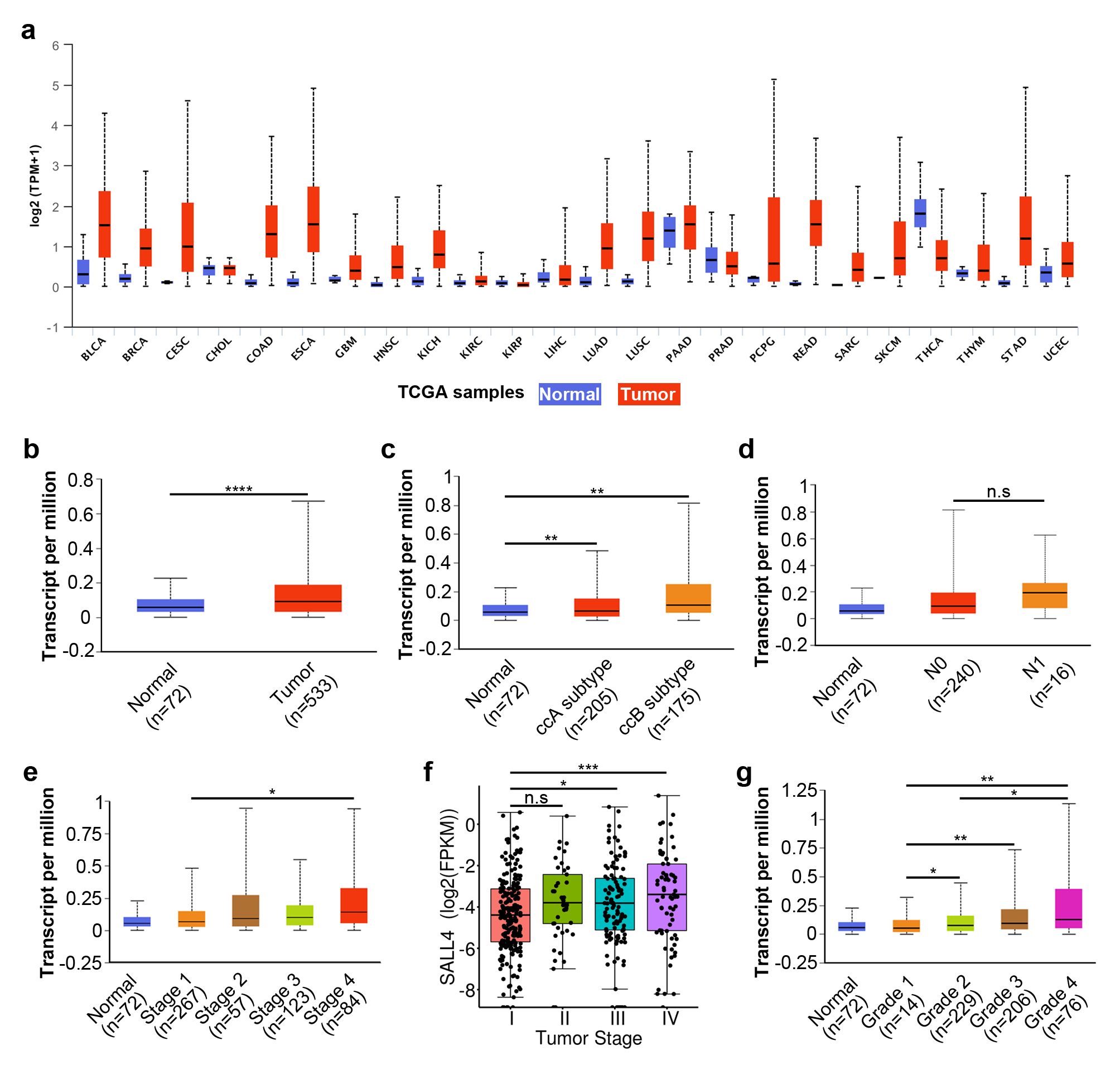

Supplement: Supplementary file 1 — Additional file 1:Figure S1. SALL4 is upregulated in ccRCC in the TCGA project. a Pan-cancer analysis for SALL4 expression in normal and tumor tissues. b Differential expression of SALL4 in normal kidney and ccRCC tissues. c Relative SALL4 expression in normal kidney and ccRCC subtypes. d-g Analysis of SALL4 expression based on lymphatic metastasis (d), AJCC stage (e, f) and histological grade (g). n.s = no significance, * P < 0.05, ** P < 0.001, *** P < 0.001 and **** P < 0.0001. [file 13046_2020_1609_MOESM1_ESM.tif]

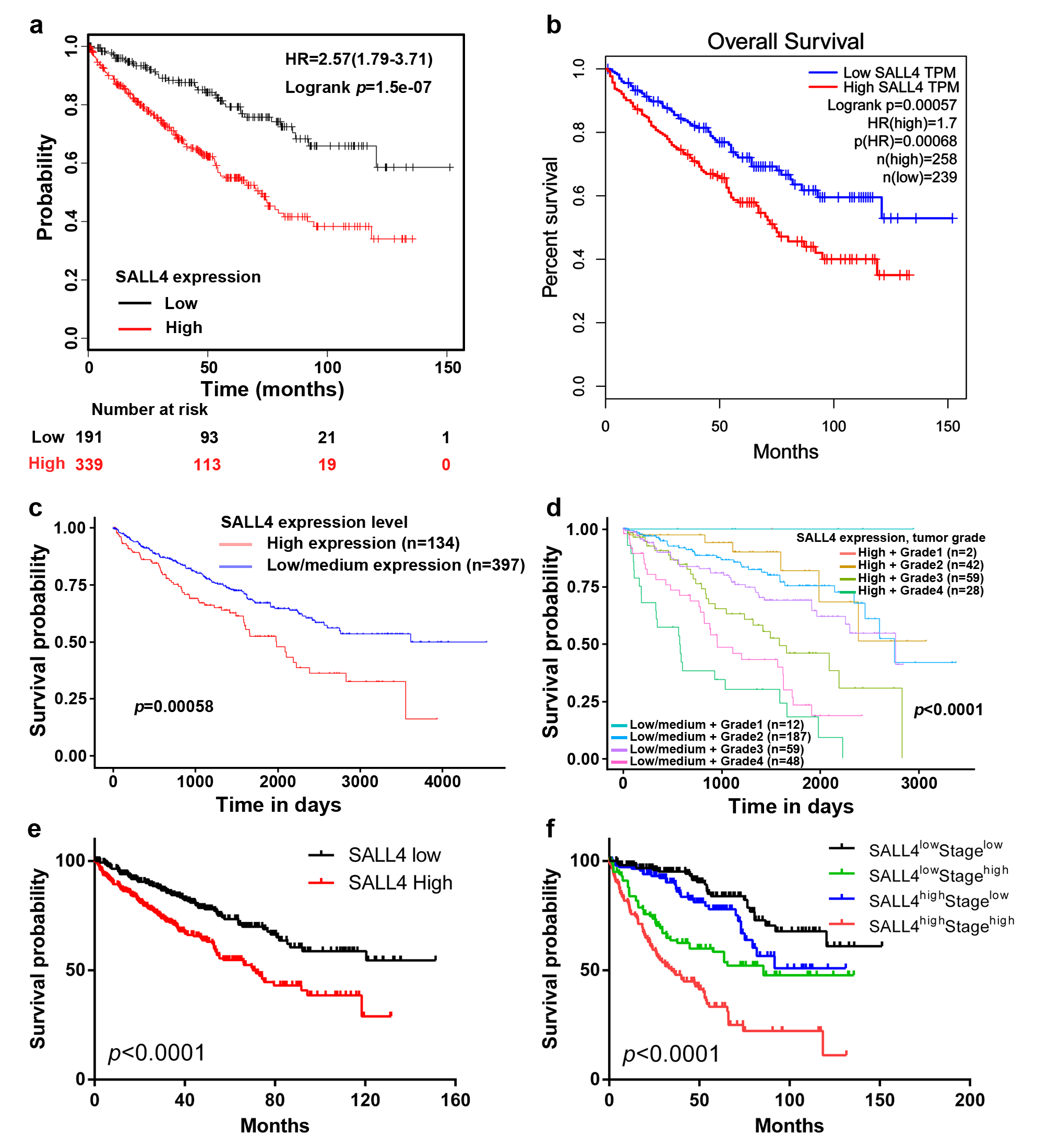

Supplement: Supplementary file 2 — Additional file 2:Figure S2. Upregulation of SALL4 predicts poor prognosis in ccRCC patients from TCGA database. a Overall survival analysis for the overall survival of ccRCC patients via Kaplan-Meier plotter. b Kaplan-Meier analysis of overall survival in ccRCC patients based on SALL4 expression via GEPIA. c, d Kaplan-Meier survival curves for ccRCC patients based on SALL4 expression and histological grade by UALCAN bioinformatics. e, f Kaplan-Meier survival analyses for ccRCC patients depending on SALL4 expression and AJCC stage via TCGAportal. [file 13046_2020_1609_MOESM2_ESM.tif]

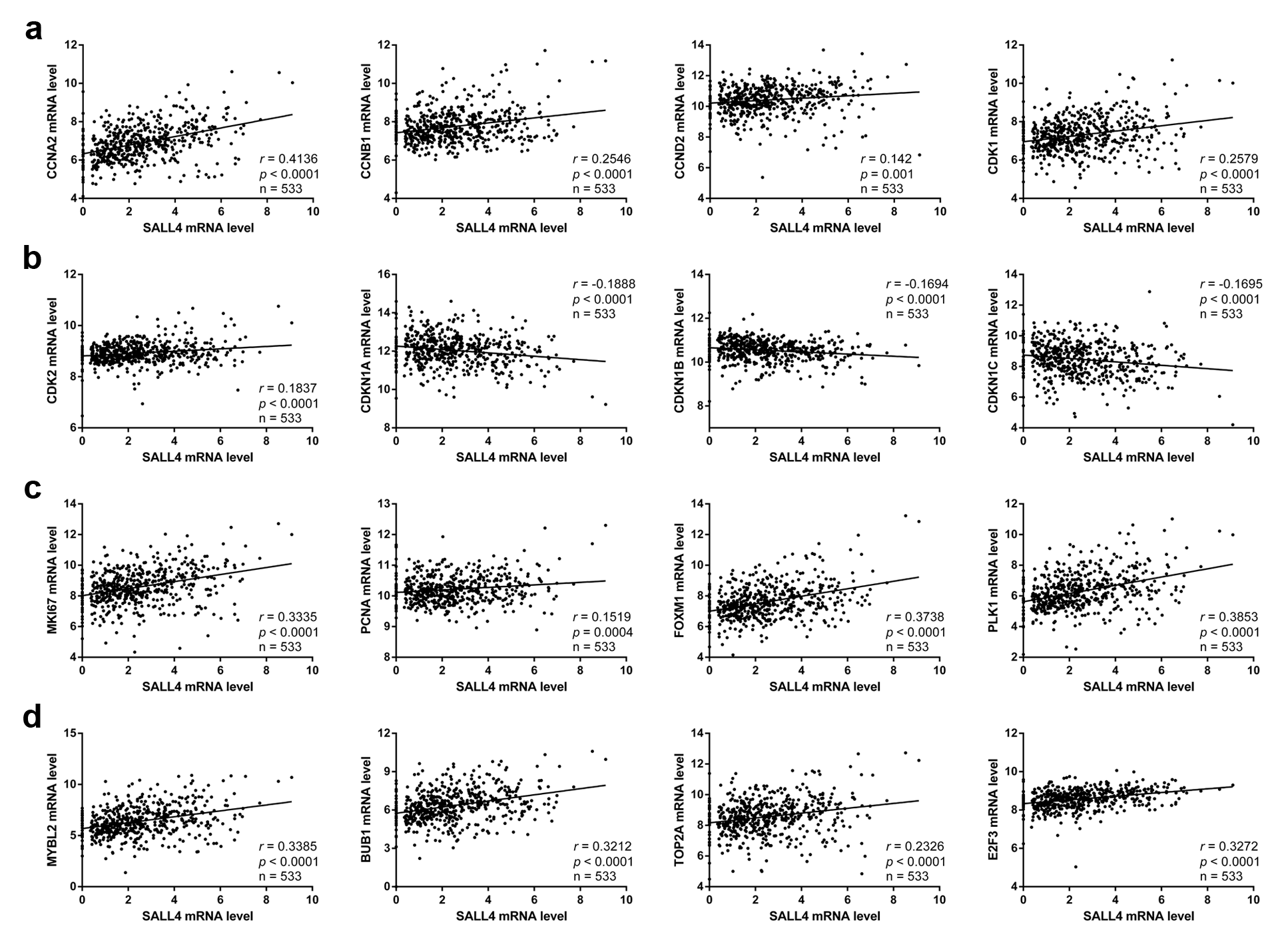

Supplement: Supplementary file 3 — Additional file 3:Figure S3. Pearson correlation analysis of SALL4 mRNA expression with the transcripts of proliferation-associated genes. Scatter plots depicting the significant correlation between SALL4 expression and the mRNA levels of CCNA2, CCNB1, CCND2, CDK1 (a), CDK2, CDKN1A, CDKN1B, CDKN1C (b), MKI67, PCNA, FOXM1, PLK1 (c), MYBL2, BUB1, TOP2A and E2F3 (d). Data were acquired from TCGA database and analyzed by LinkedOmics. [file 13046_2020_1609_MOESM3_ESM.tif]

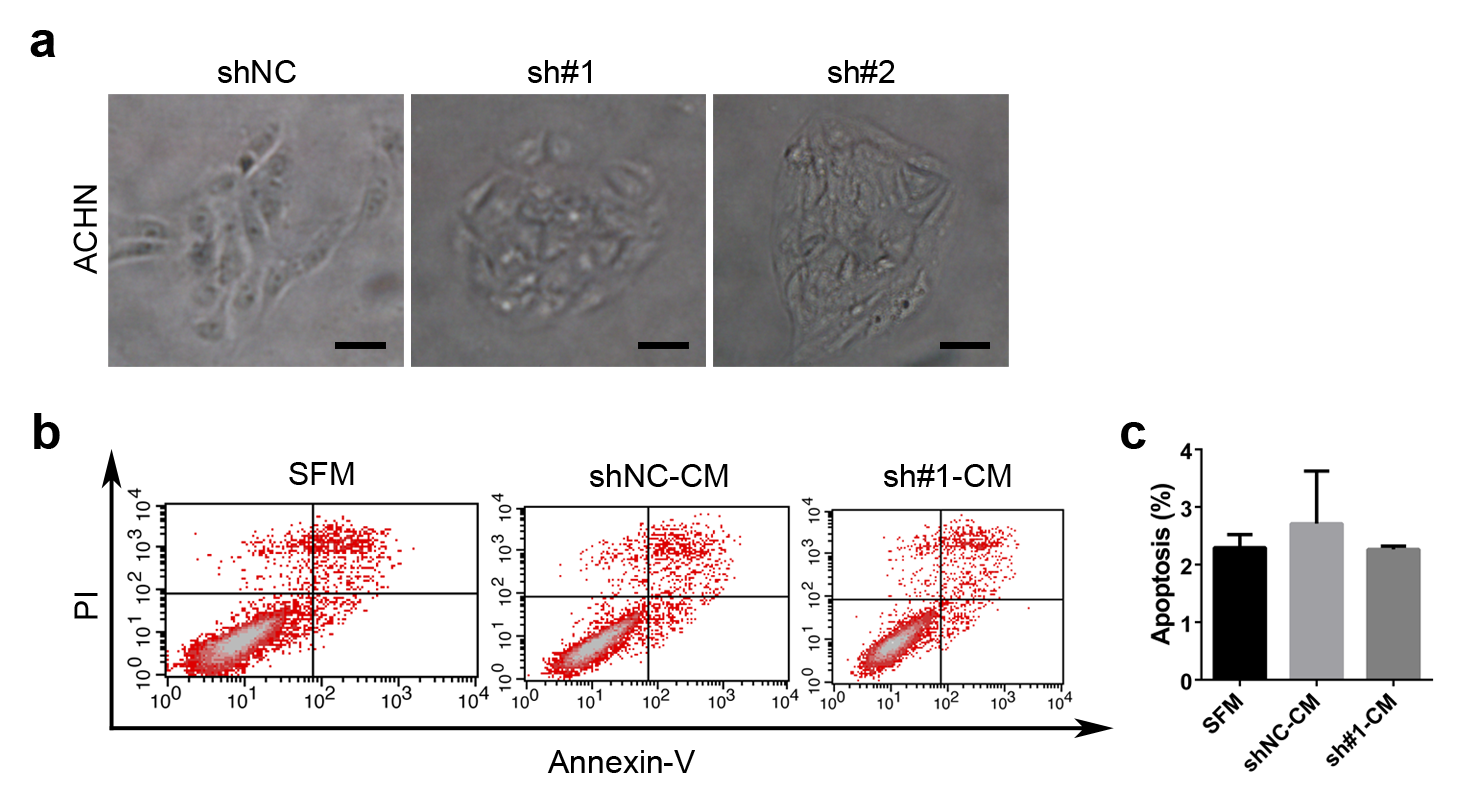

Supplement: Supplementary file 5 — Additional file 5:Figure S4.a Morphological observation of ACHN cells with indicted shRNA treatment under light microscope (scale bar, 50 μm). b Flow cytometry analysis of apoptosis in HUVECs treated with indicted SFM or CMs. c Quantification analysis of cell apoptosis in HUVECs with indicted treatment. [file 13046_2020_1609_MOESM5_ESM.tif]

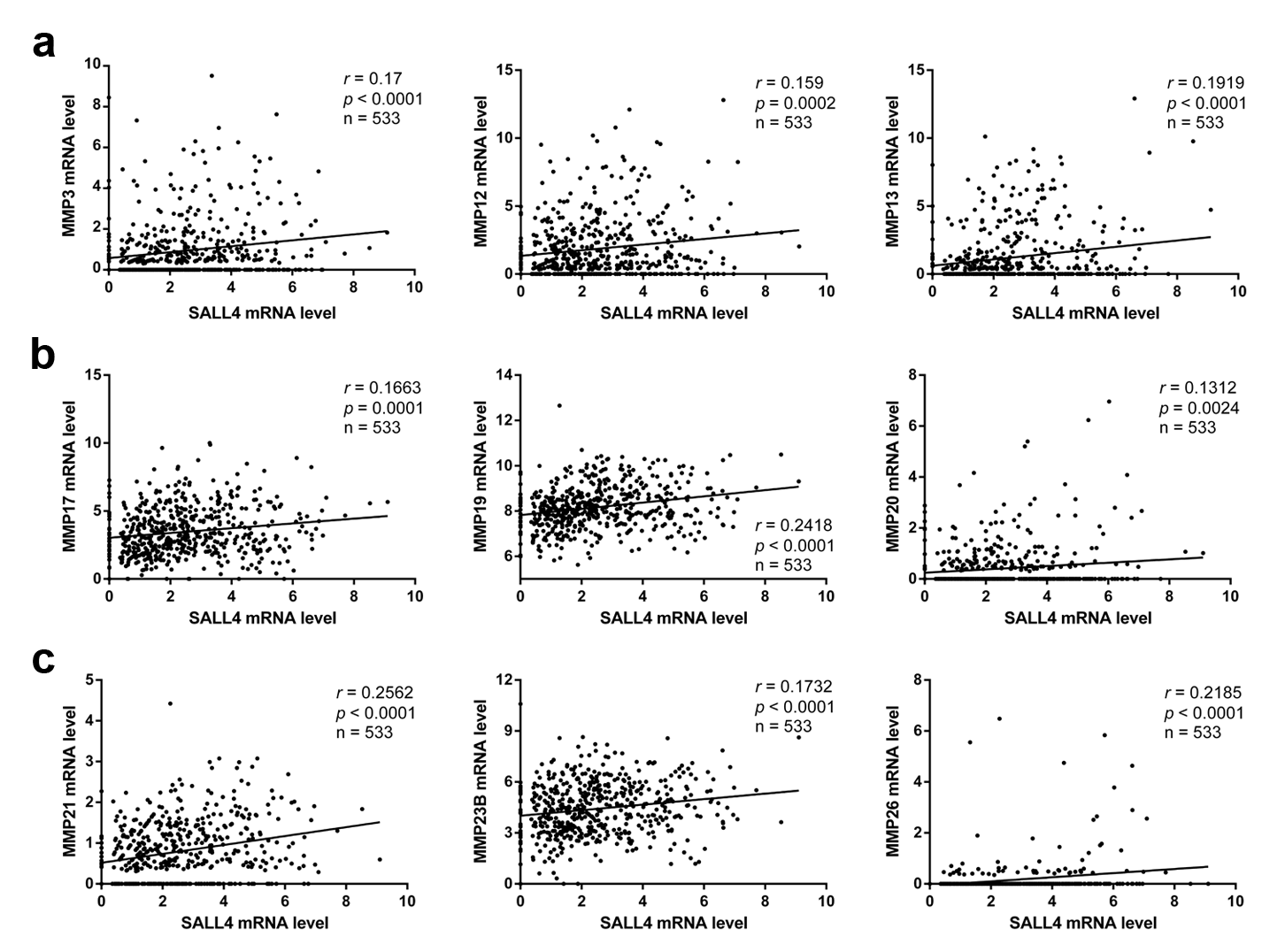

Supplement: Supplementary file 6 — Additional file 6:Figure S5. Pearson correlation analysis of SALL4 mRNA expression with the transcripts of matrix metalloproteinases (MMPs). Scatter plots depicting the significant correlation between SALL4 expression and the mRNA levels of MMP3, MMP12, MMP13 (a), MMP17, MMP19, MMP20 (b), MMP21, MMP23B and MMP26 (c). Data were acquired from TCGA database and analyzed by LinkedOmics. [file 13046_2020_1609_MOESM6_ESM.tif]

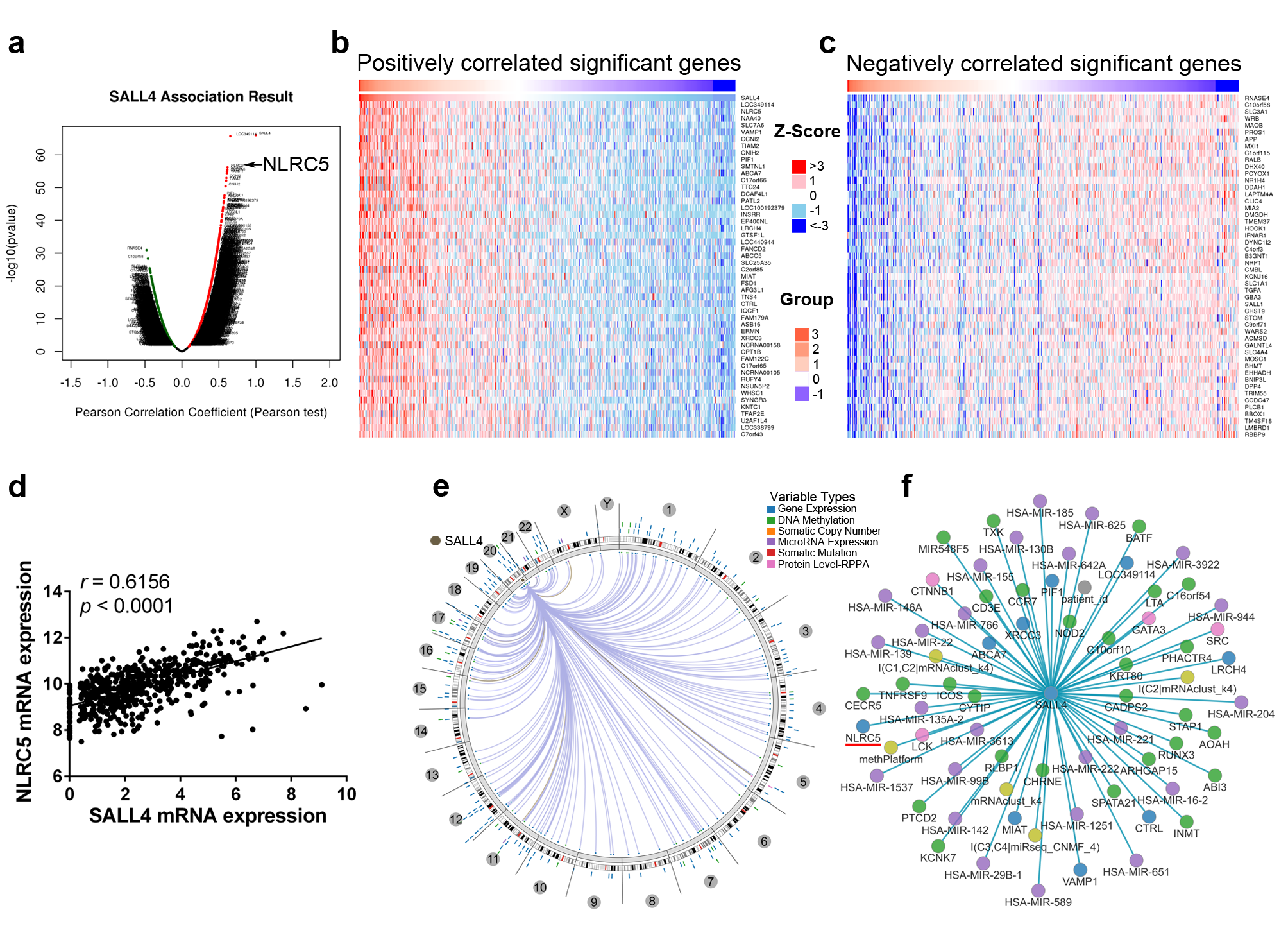

Supplement: Supplementary file 8 — Additional file 8:Figure S6. Integrative multi-omics analysis of SALL4 mRNA expression in ccRCC. a Volcano plot of Pearson correlation coefficient analysis of SALL4 with genes differentially expressed in ccRCC. Red (green) dots represent genes positively (negatively) correlated with SALL4. b, c The heat maps showing the top 50 genes exhibiting significant positive (b) and negative (c) correlation with SALL4 in ccRCC. d Pearson correlation of SALL4 gene expression with NLRC5 transcript in ccRCC patients. Data (a-d) from TCGA database were analyzed via LinkedOmics bioinformatics. e Genome-wide association of SALL4 mRNA expression with multifarious molecular features in ccRCC. The arcs connected pairs of dots representing the features to indicate statistically significant associations. f The significant correlation between SALL4 gene expression with multiple molecular features were visualized using network plot. Data (e, f) from TCGA database were analyzed using Regulome Explorer. [file 13046_2020_1609_MOESM8_ESM.tif]

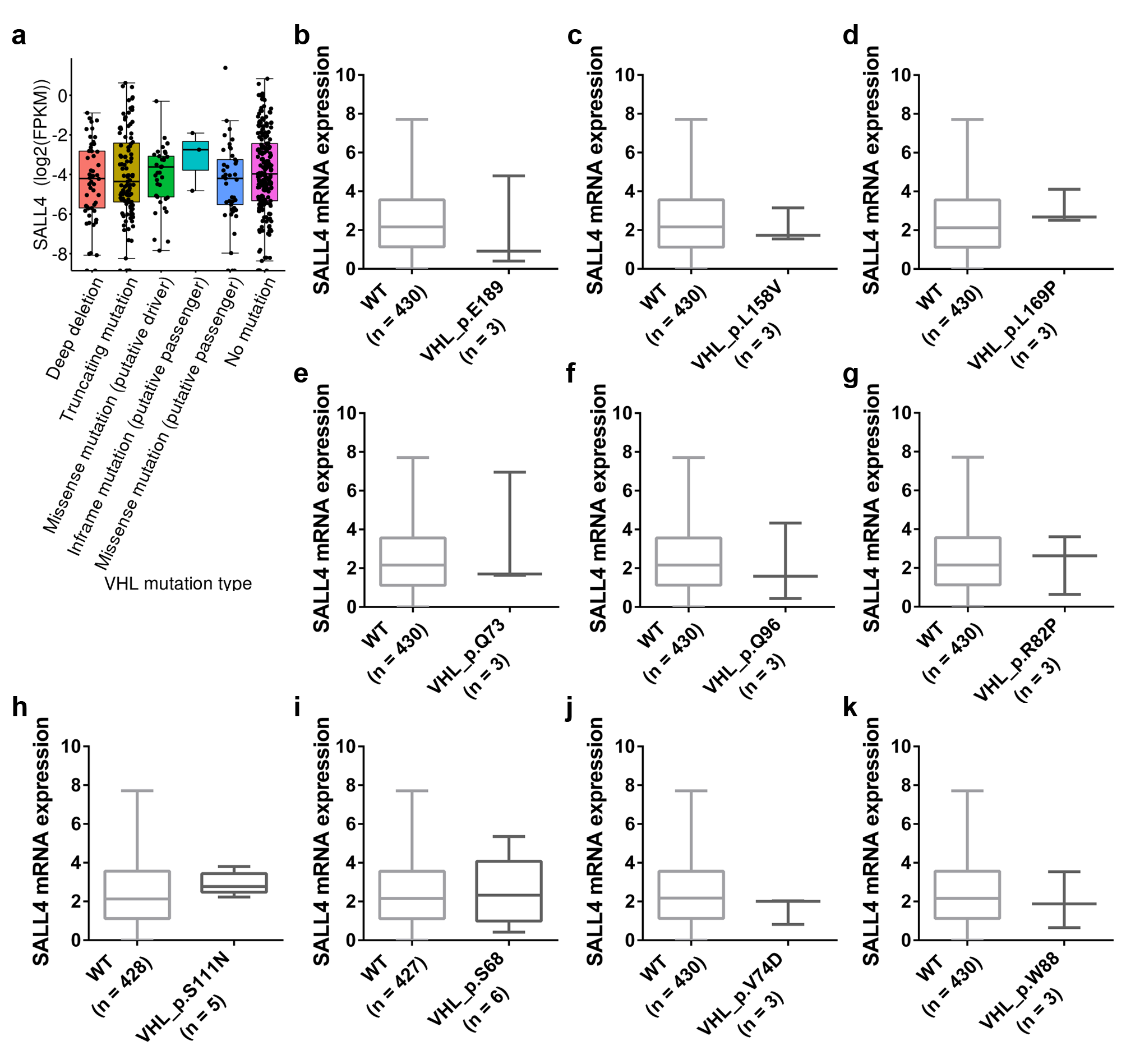

Supplement: Supplementary file 9 — Additional file 9:Figure S7.. Functional impact of VHL mutation on SALL4 mRNA expression in ccRCC patients. a Analysis of SALL4 expression difference among ccRCC patients with indicated VHL mutation types. Data were acquired from TCGA database and analyzed by TCGAportal. b-k Association between SALL4 mRNA expression and VHL point mutations in ccRCC patients. Data were acquired from TCGA database and analyzed by LinkedOmics. [file 13046_2020_1609_MOESM9_ESM.tif]

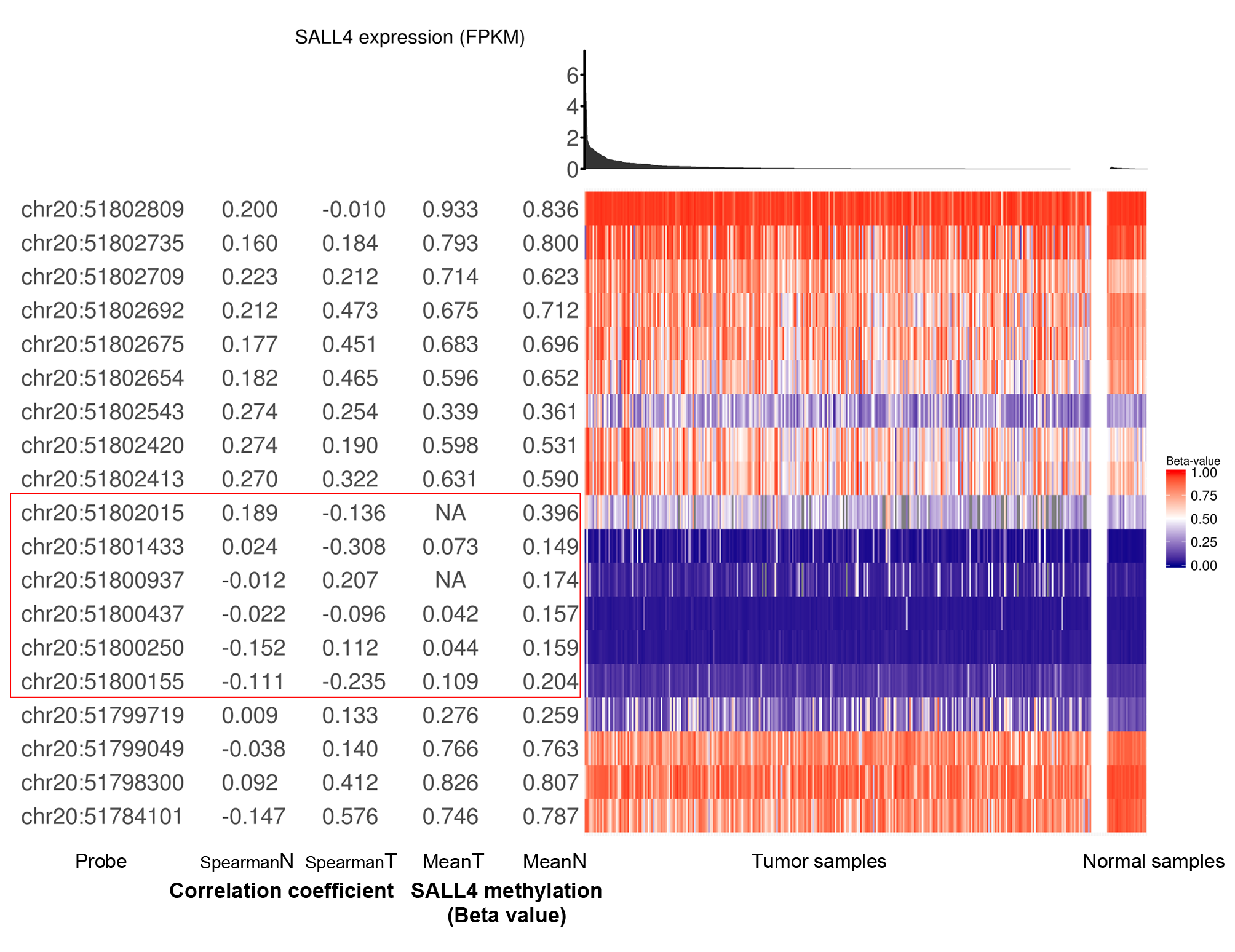

Supplement: Supplementary file 10 — Additional file 10:Figure S8.. Association analysis of SALL4 gene expression with its probe methylation in primary ccRCC tumor and matched normal tissues. Data were analyzed by TCGAportal. [file 13046_2020_1609_MOESM10_ESM.tif]

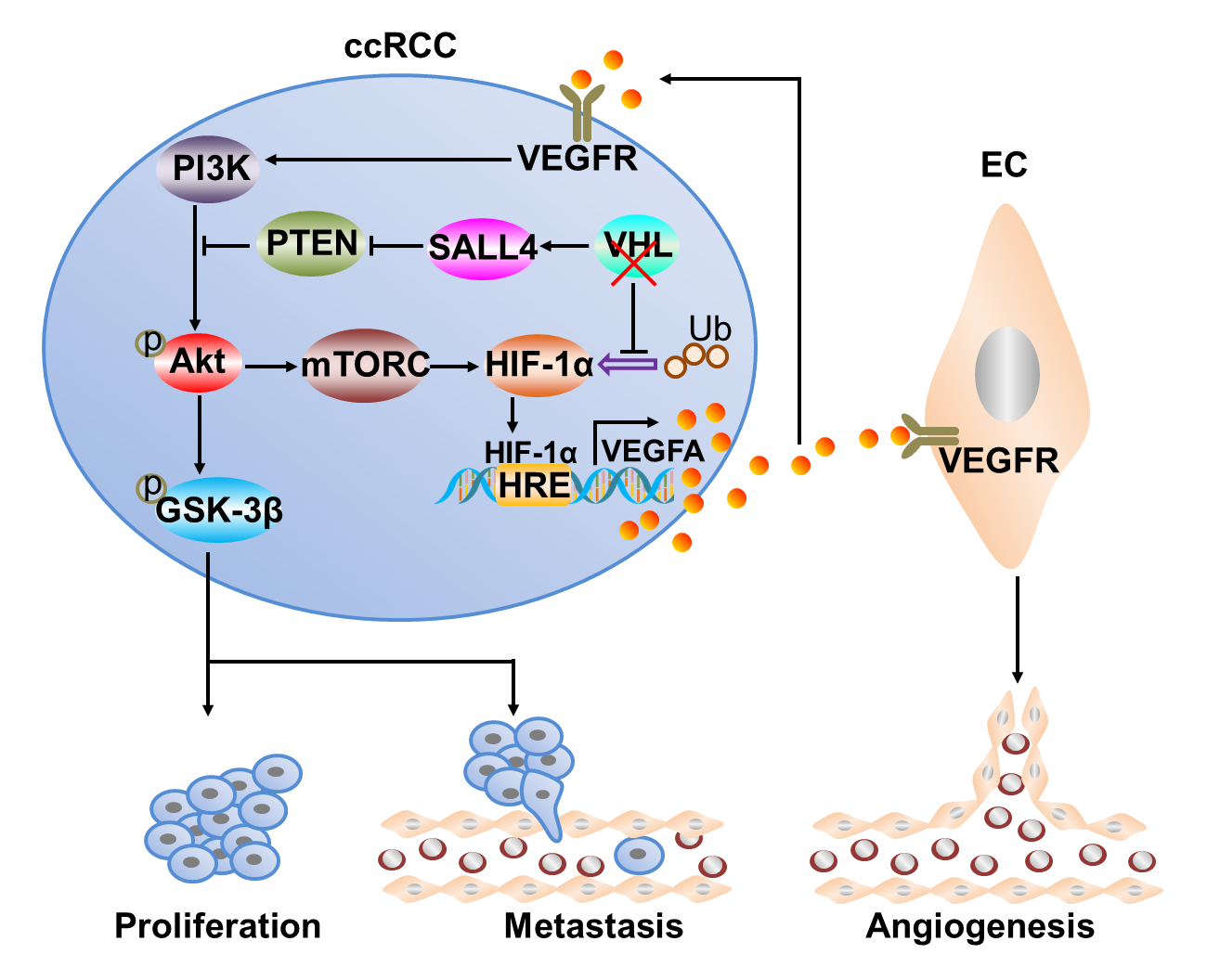

Supplement: Supplementary file 11 — Additional file 11:Figure S9.. The schematic diagram for elevated SALL4-mediated tumorigenesis and angiogenesis in ccRCC. Ub, ubiquitin; EC, endothelial cell; mTORC, mTOR complex; HRE, hypoxia response element. [file 13046_2020_1609_MOESM11_ESM.tif]
