# Supplementary material for: VHL mutation-mediated SALL4 overexpression promotes tumorigenesis and vascularization of clear cell renal cell carcinoma via Akt/GSK-3β signaling
Source: J Exp Clin Cancer Res. 2020 Jun 8;39:104. doi: 10.1186/s13046-020-01609-8 (PMC7278163; doi:10.1186/s13046-020-01609-8)
Supplement: Supplementary file 4 — Additional file 4:Table S1. Correlation between SALL4 and proliferation-associated genes. [file 13046_2020_1609_MOESM4_ESM.docx]

**Table S1** Correlation between SALL4 and proliferation-associated genes

| Target Gene | Pearson Correlation | *P*-value | FDR (BH) |
| --- | --- | --- | --- |
| CCNA1 | 3.332e-01 | 2.739e-15 | 3.872e-14 |
| CCNA2 | 4.136e-01 | 1.937e-23 | 8.977e-22 |
| CCNB1 | 2.546e-01 | 2.496e-09 | 1.430e-08 |
| CCNB2 | 3.332e-01 | 2.749e-15 | 3.883e-14 |
| CCND2 | 1.420e-01 | 1.014e-03 | 2.225e-03 |
| CCNE1 | 4.145e-01 | 1.507e-23 | 7.146e-22 |
| CCNE2 | 2.046e-01 | 1.906e-06 | 6.823e-06 |
| CDK1 | 2.579e-01 | 1.525e-09 | 9.037e-09 |
| CDK2 | 1.837e-01 | 1.987e-05 | 5.972e-05 |
| CDK3 | 3.811e-01 | 7.187e-20 | 1.914e-18 |
| CDKN1A | -1.888e-01 | 1.143e-05 | 3.582e-05 |
| CDKN1B | -1.694e-01 | 8.505e-05 | 2.295e-04 |
| CDKN1C | -1.695e-01 | 8.385e-05 | 2.266e-04 |
| E2F1 | 3.302e-01 | 5.088e-15 | 6.884e-14 |
| E2F2 | 3.502e-01 | 7.938e-17 | 1.376e-15 |
| E2F3 | 3.272e-01 | 9.162e-15 | 1.199e-13 |
| E2F4 | 3.942e-01 | 2.942e-21 | 9.675e-20 |
| E2F7 | 3.417e-01 | 4.852e-16 | 7.577e-15 |
| E2F8 | 2.784e-01 | 6.069e-11 | 4.465e-10 |
| RB1 | -3.032e-01 | 8.459e-13 | 8.262e-12 |
| MKI67 | 3.335e-01 | 2.592e-15 | 3.680e-14 |
| PCNA | 1.519e-01 | 4.320e-04 | 1.019e-03 |
| PLK1 | 3.853e-01 | 2.596e-20 | 7.339e-19 |
| FOXM1 | 3.738e-01 | 4.008e-19 | 9.677e-18 |
| BUB1 | 3.212e-01 | 2.941e-14 | 3.554e-13 |
| MYBL2 | 3.385e-01 | 9.407e-16 | 1.418e-14 |
| TOP2A | 2.326e-01 | 5.569e-08 | 2.545e-07 |
