# Supplementary material for: VHL mutation-mediated SALL4 overexpression promotes tumorigenesis and vascularization of clear cell renal cell carcinoma via Akt/GSK-3β signaling
Source: J Exp Clin Cancer Res. 2020 Jun 8;39:104. doi: 10.1186/s13046-020-01609-8 (PMC7278163; doi:10.1186/s13046-020-01609-8)
Supplement: Supplementary file 7 — Additional file 7:Table S2. Association of SALL4 with matrix metalloproteinases (MMPs) and tissue inhibitors of metalloproteinases (TIMPs). [file 13046_2020_1609_MOESM7_ESM.docx]

**Table S2** Association of SALL4 with matrix metalloproteinases (MMPs) and tissue inhibitors of metalloproteinases (TIMPs)

| Target Gene | Pearson Correlation | *P*-value | FDR (BH) |
| --- | --- | --- | --- |
| MMP3 | 1.700e-01 | 7.973e-05 | 2.162e-04 |
| MMP9 | 1.227e-01 | 4.552e-03 | 8.742e-03 |
| MMP12 | 1.590e-01 | 2.290e-04 | 5.687e-04 |
| MMP13 | 1.919e-01 | 8.108e-06 | 2.617e-05 |
| MMP17 | 1.663e-01 | 1.147e-04 | 3.023e-04 |
| MMP19 | 2.418e-01 | 1.569e-08 | 7.871e-08 |
| MMP20 | 1.312e-01 | 2.410e-03 | 4.900e-03 |
| MMP21 | 2.562e-01 | 1.969e-09 | 1.142e-08 |
| MMP23B | 1.732e-01 | 5.812e-05 | 1.616e-04 |
| MMP25 | 2.601e-01 | 1.086e-09 | 6.585e-09 |
| MMP26 | 2.185e-01 | 3.494e-07 | 1.408e-06 |
| TIMP3 | -2.221e-01 | 2.219e-07 | 9.218e-07 |
| TIMP4 | -2.761e-01 | 8.773e-11 | 6.303e-10 |
